# Untargeted approaches based on GC-Orbitrap-HRMS and two-dimensional GC–MS for the identification of intentionally and non-intentionally added substances from bio-based food contact materials

**DOI:** 10.1007/s00216-026-06444-y

**Published:** 2026-03-26

**Authors:** Maurizio Piergiovanni, Simone Squara, Marco Fontanarosa, Cristian Maffezzoni, Nicolò Riboni, Antonella Cavazza, Monica Mattarozzi, Federica Bianchi, Michele Suman, Maria Careri

**Affiliations:** 1 University of Parma, Department of Chemistry, Life Sciences and Environmental Sustainability, Parco Area Delle Scienze 17/A, 43124 Parma, Italy; 2 Barilla G.R. F.lli SpA, Research, Development & Quality, Sensory and Analytical Food Science, Via Mantova 166, 43122 Parma, Italy; 3 University of Parma, Department of Food and Drug, Parco Area delle Scienze 27/A, 43124 Parma, Italy

**Keywords:** GC-Orbitrap HRMS, GC × GC-TOF–MS, Bioplastic, Food contact materials, NIAS, PLA

## Abstract

The increasing adoption of bioplastics is rapidly expanding in food contact materials (FCMs) to minimise environmental impact and improve consumers’ acceptance. However, several aspects must be considered throughout their lifecycle, from bioplastic processing to final product application. This study presents an untargeted analytical workflow to characterise volatile compounds of bioplastic FCMs based on GC–Orbitrap HRMS coupled with chemometrics and complemented by GC × GC-TOF–MS. The bio-based FCMs included polylactic acid (PLA), its heat-resistant variant crystallised PLA (cPLA) and no-PLA materials. The approach based on GC-Orbitrap HRMS coupled to supervised PLS-DA and variable importance in projection (VIP) method allowed the identification of differential compounds able to discriminate samples according to material composition and maximum end-use temperature. Chemometric analysis applied to samples classified according to maximum end-use temperature identified cyclic oligomers and hydrocarbons as markers for PLA samples suitable for use up to 40°C. Considering classification by the type of material resistant to moderate temperature (cPLA *vs.* no-PLA FCM), analysis of the dataset showed almost completely different VIP selection, with some PLA-related molecules (lactic acid, lactate esters, and linear oligomers) characterising cPLA, some hydrocarbons as key variables of the non-PLA class, and two succinate esters present in both the materials suggesting the presence of PLA/polybutylene succinate blends. In addition to polymer-related molecules, plasticisers emerged as chemical additives incorporated into the investigated materials during manufacturing. In addition, GC × GC-TOF–MS successfully identified distinct series of PLA oligomers, citric acid esters, and mineral oil saturated hydrocarbons, with regular chromatographic patterns, supporting structural hypotheses and integrating GC–Orbitrap HRMS findings.

**Graphical Abstract:**

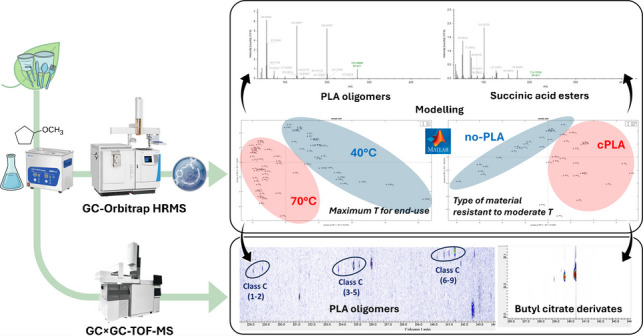

**Supplementary Information:**

The online version contains supplementary material available at 10.1007/s00216-026-06444-y.

## Introduction

The use of bioplastics is rapidly spreading in the world of food contact materials (FCMs) to reduce the environmental footprint, increase cost competitiveness and improve consumers’ acceptance [1]. This transition complies with the circular economy goals and with Directive (EU) 2019/904 on the reduction of single-use plastic products [2]. Additionally, high production cost and poor scalability remain a challenge along with regulatory and market barriers. Biobased materials designed to replace single-use plastics are frequently complex formulations rather than simple, pure natural polymers [3, 4]. While promoted as sustainable, these materials often suffer from a lack of ingredient transparency, with manufacturers providing only general descriptions of their bio-based origin. Therefore, even though bio-based items are well accepted by consumers and are perceived as safe materials, the absence of harmful substances must be investigated by developing reliable analytical methods and applying appropriate risk assessment procedures, as suggested by Zimmermann et al. [5]. Although the United Nations Sustainable Development Goals have been established and many companies have promoted sustainable practices, the transition to eco-friendly plastics is still slow and requires country-specific policies [6]; moreover, the transition remains uneven as regulatory frameworks and implementation practices differ across regions, resulting in heterogeneous market uptake and compliance approaches. As a result, currently no general methodologies and harmonised rules are available that are specifically designed to assess the compliance of bioplastic FCMs.

In this context, innovative analytical strategies are required to determine both intentionally added substances (IAS) and non-intentionally added substances (NIAS). In particular, the development of untargeted strategies for the characterisation of the material composition and the identification of migrating compounds is required [7, 8]. To this end, it is worth highlighting that the use of methodologies that comply with the principles of green analytical chemistry (GAC) [9] represents an added value to minimize the environmental impact and guarantee the operator safety.

The development of untargeted analytical strategies able to investigate IAS, predictable NIAS, and unexpected NIAS is strongly encouraged for a deep characterisation of bioplastics, taking into account that the identification of unknown NIAS in plastic food packaging is challenged by the chemical complexity of the materials, the lack of reference standards, and the high sensitivity required to detect low-level concentration [7]. In this context, advanced analytical platforms are often required because NIAS may be present at trace levels within complex mixtures of additives, degradation products, and processing-related contaminants. Under these conditions, conventional one-dimensional GC–MS can be limited by co-elution and matrix interferences, which may hinder both detection and reliable annotation in untargeted screening**.** Therefore, approaches providing enhanced selectivity (*e.g.*, accurate-mass filtering in GC–HRMS) and/or enhanced separation capacity (e.g., orthogonal separations in GC × GC) are critical to support compound detection and prioritisation in chemometric workflows. Analytical techniques offering enhanced separation and/or detection capabilities, as hyphenated mass spectrometry (MS)-based platforms, are needed to address this analytical challenge. Since IAS and NIAS can be volatile, semi-volatile or non-volatile, their comprehensive assessment benefits from strategies that combine liquid chromatography (LC) and gas chromatography (GC)-MS based techniques [10, 11], appropriate sample treatment procedures [12], as well as multidimensional chromatography separations (GC × GC, 2D LC, LC-GC) [13–18]. High-resolution (HR) mass analysers, such as Orbitrap and QTOF, enable a high level of confidence in the identification of non-targeted analytes, even in the absence of reference standards [19–21]. Orbitrap technology provides high sensitivity, making it effective for detecting NIAS at low concentrations, often in the low µg/kg range, such as in food safety analysis [22]. The combined use of GC × GC and GC-HRMS has been also demonstrated in a previous study exploring the potential of different GC–MS-based approaches, including GC-Orbitrap-HRMS and GC × GC-TOF-low resolution (LR)MS, for the identification of novel NIAS extracted from two polyester-polyurethane lacquers with acetonitrile [18]. It should be noted that in addition to the limited availability of standards, many IAS and NIAS are missing from mass databases and spectral libraries, resulting in a significant number of compounds remaining unidentified. However, some related compounds can be reliably recognised and attributed to a chemical class based on characteristic ions or ion series using HRMS, as in the case of oligomers and hydrocarbon isomers. Biederman et al. have discussed how signals showing systematic order and regularity in the GC × GC plot may be ascribed to related components of FCMs, facilitating their identification. Moreover, owing to the structured two-dimensional separation space, compounds that do not follow the dominant distribution patterns in the GC × GC plane can be more readily recognised as chemically distinct components [14]. Finally, modulation can provide signal focusing, increasing peak height and improving signal-to-noise, which can help detect low-abundance compounds.

For untargeted analysis, integrating MS-feature extraction and annotation with multivariate chemometric analysis into the analytical workflow is required to extract useful information from complex dataset. In the context of FCM characterisation, this approach allows prioritisation of MS-features associated with a particular sample category, such as polymer type and conditions of use, supporting data interpretation [23].

Very recently, we developed a novel extraction method using the green solvent cyclopentyl methyl ether (CPME) to increase the sustainability of analytical workflows [24]. A central composite design of experiments was applied to optimise the ultrasound-assisted extraction (UAE) of volatile and semi-volatile components from biobased tableware samples, which were subsequently identified by GC-(LR)MS.

In this context, the present research aims to explore the complementarity of two cutting-edge analytical techniques, namely GC-Orbitrap HRMS and GC × GC–MS, evaluating the potential in their combined use. In particular, the study focuses on both the potential of high-resolution MS coupled to chemometrics for univocal identification and structural elucidation of compounds in biobased FCMs and the enhanced chromatographic performance of two-dimensional GC (2D-GC) to facilitate a thorough characterisation of the chemical composition of biobased materials and the identification of a larger number of compounds. To the best of our knowledge, this is the first time a GC-Orbitrap HRMS system has been investigated for the untargeted separation and detection of IAS and NIAS from commercial bioplastics. Furthermore, this research, unlike most previous work focused on the identification of IAS and NIAS, aims to explore the substances that characterise the differences between bio-based food contact materials. This approach could be useful to provide a new tool for discriminating different biobased items depending on their compositions.

## Experimental section

### Materials and reagents

CPME (99.9% purity) was purchased from Carlo Erba Reagents (Milan, Italy). Hexane, acetone, and methanol were purchased from Merck (Milan, Italy) at GC grade purity level. A C7 – C40 saturated alkanes mixture, 1000 µg/mL each component in hexane (certified reference material) was purchased from Merck and used for the determination of the linear retention indices (RI).

To prevent contamination from plastic labware (which may be a source of plastic additives), glassware or stainless-steel apparatus were used. Prior to use, all glass apparatus was soaked in 10% HCl for 12 h, rinsed with Milli-Q water, maintained at 140 °C for 6 h, and rinsed with methanol.

### Samples

A total of 25 biobased FCMs commercially available from different manufacturing companies were purchased to represent different bio-based material types, i.e. PLA, crystallised PLA (cPLA) and other bioplastic materials. This sample set included cutleries, glasses, coffee sticks, straws, and cups [24]. According to the information reported on the label, samples were classified into categories based on the material: PLA, cPLA, and no-PLA, and indications of the maximum temperature for end-use (70°C for 2 h; 40 °C for 2 h). No-PLA category is reasonably expected to be made of different biopolymers, such as polybutylene succinate (PBS), or blends obtained by combining biopolymers with polypropylene (PP) at various percentages. Material characterisation was carried out by attenuated total reflection–Fourier transform infrared spectroscopy (ATR-FTIR) and differential scanning calorimetry (DSC), as described in the previous work [24]. The list of samples is reported in Table S1.

### Sample preparation

Samples were first ground using an A11 basic analytical mill (IKA, Staufen, Germany) in the presence of liquid nitrogen to avoid degradation of materials. The sample preparation procedure was based on a previously optimised method that was developed to improve extraction of analytes using a green solvent [24]. Briefly, 0.2 g of sample was added with 4 mL of CPME and extracted in mixed mode by liquid–liquid extraction for 9h:30 min coupled to UAE using an ultrasonic bath (J.P. Selecta, Barcelona, ​​Spain) at room temperature and 100% power for 25 min. The final extracts were centrifuged, filtered through 0.2 µm PTFE syringe filters, split into aliquots and stored at −80°C before the instrumental analysis by GC-Orbitrap HRMS and GC × GC–MS. Three independent extractions were performed for each item.

Quality control samples (pooled QCs) were prepared by mixing equal volumes (500 µL) of each extract.

### GC-Orbitrap HRMS analysis

GC-Orbitrap HRMS analysis was performed using a Thermo Scientific™ TRACE™ 1610 Series Gas Chromatograph coupled to a Thermo Scientific™ Orbitrap™ Exploris™ GC 240 Mass Spectrometer.

Chromatographic separation was performed using a 30 m × 0.25 mm i.d., 0.25 μm thickness Thermo Scientific™ TraceGOLD TG-5SilMS GC capillary column, using He as carrier gas (flow 1.2 mL/min) under the following oven temperature program conditions: 60 °C for 2 min, 8 °C/min to 280 °C, holding the final temperature for 5 min. The transfer line was maintained at 280 °C. The injector was operated at 270 °C in split mode (split ratio 1:1) and the injection volume was 1 µL.

Electron ionisation (EI) was performed at 70 eV with the ion source operated at 280 °C. Full scan MS acquisition was carried out in profile-mode in the *m/z* 35–500 range. The Orbitrap resolving power was set at 60,000 FWHM at *m/z* 200. The automatic gain control (AGC) target was set at 1 × e^6^ with a maximum injection time (IT) of 200 ms. Mass calibration was performed daily using perfluorotributylamine (FC-43) as calibrating gas. Internal mass calibration was performed by using the *m/z* 207.03235 background ion C_5_H_15_O_3_Si_3_^+^ from the column bleed as lock mass with a search window of ± 2 ppm.

Thermo Scientific™ Xcalibur™ v. 4.5 software was used for signal acquisition. QCs were analysed in duplicate after every fifth injection of sample extracts to correct for any variability or drift in instrument performance throughout the analytical run.

### GC × GC–MS analysis

GC × GC–MS analysis was carried out using an Agilent 8890 GC instrument (Agilent Technologies, Little Falls, DE) incorporating a FLUX™ flow modulator (LECO Corporation, St. Joseph, MI) and coupled with a LECO Pegasus® BT 4D mass spectrometer (LECO Corporation, St. Joseph, MI). Liquid injection was carried out using a L-PAL3 system (CTC Analytics AG, Zwingen, CH) with an injection volume of 2 µL. The injector temperature was held at 270 °C, operating in splitless mode.

The column settings and operative conditions were as follows: first dimension (1D) column Rxi-5Sil MS ((5%-phenyl)-methylpolysiloxane, 30 m × 0.25 mm d_c_ × 0.50 μm d_f_); second dimension (2D) column Rxi-17Sil MS [equivalent to (50%-phenyl)-methylpolysiloxane 1.0 m × 0.1 mm d_c_ × 0.10 μm d_f_] from Restek S.r.l., of which the last 0.31 m constituted the transfer line. The first 0.69 m of the ^2^D column was placed in a secondary oven with a temperature offset of + 5 °C. The modulation period (P_M_) was 1.2 s and the valve remained in the injection position for 80 ms. The P_M_ was selected to provide adequate 1D peak sampling (≥ 3 modulated slices per 1D peak, *i.e.*, modulation ratio MR ≥ 3). The carrier gas was helium, which was maintained at a ^1^D flow rate of 1.2 mL min^−1^ in the constant flow mode and at a ^2^D flow rate of 3.5 mL min⁻^1^. The oven temperature ramp was: 40 °C (0.2 min) to 250 °C (2.0 min) at 5.0 °C min^−1^.

The acquisition parameters consisted of an acquisition rate of 200 Hz within the *m/z* 45 − 550 range after a solvent delay of 600 s; the detector was set to the maximum sensitivity parameter. The ion source and transfer line temperatures were both set at 270 °C.

Compound identification was verified using the NIST 23 library (acceptance criteria: a spectral similarity value higher than 750 and a ΔRI ≤ 20 (ΔRI as difference between the experimental and tabulated RI).

QCs were analysed in duplicate after every tenth injection of sample extracts to correct for any ^1^D shifts or drift in instrument performance throughout the analytical run. A blank sample was positioned after every QC sample for carry-over monitoring. All the samples were analysed in a single analytical batch. GC × GC–MS runs were acquired using LECO ChromaTOF® software v 1.2.0.6, then exported to netCDF, and processed using GC Image MDC Software, V2025r2 (GC Image LCC, Lincoln, Nebraska).

### Untargeted workflow and multivariate analysis

Thermo Scientific™ Compound Discoverer™ software (CD, v. 3.3 SP3, ThermoFisher Scientific, Waltham, MA) was used to perform data processing including spectral deconvolution, retention time alignment, background removal, feature filtering and spectral library search for compound annotation based on the NIST 2017 mass spectral library. Details of the CD Workflow Tree are reported in Fig. S1.

As for multivariate statistics, unsupervised data analysis (Principal Component Analysis, PCA) and supervised modelling (Partial Least Square-Discriminant Analysis, PLS-DA) were performed on the autoscaled data using the PCA toolbox [25] and the classification toolbox [26] for MATLAB, respectively.To identify significant features for differentiating the sample classes, CD data processing and multivariate statistics were integrated as schematised in the flowchart reported in Fig. S2. After the feature filtering (additional details are reported in the Supplementary Information, paragraph “GC-Orbitrap HRMS data filtering”), PCA exploratory data analysis was carried out on the filtered dataset to remove outliers not complying with both Q residuals and T^2^ Hotelling diagnostics. The number of PCs to be included in the model was selected using a venetian-blinds k-fold cross-validation (k = 5) and applying the Average Eigenvalue Criterion over the scree-plot. After outlier removal, the dataset was split into training and test set with the ratio of 70:30 ratio and investigated by PLS-DA. For the end-use binary classification (40 °C and 70 °C), the dataset was split into a training set of 17 samples (7 for the 40 °C class, and 10 for the 70 °C class) and a test set of 7 samples (2 for the 40 °C class, and 5 for the 70 °C class). For the binary classification by material type resistant to moderate temperatures (cPLA and no-PLA), a subset of data corresponding to the 70 °C condition of use was split into a training set of 10 samples (5 cPLA and 5 no-PLA) and a test set of 4 samples (2 cPLA and 2 no-PLA). In both cases, the number of variables was 3. The PLS-DA models were calculated on the training set, and the optimal number of latent variables was selected by venetian-blinds k-fold cross-validation (k = 5) based on minimization of the prediction error-rate, using the maximum variance assignation criteria. Then, the model was externally validated using the test set, After that, the calculation of the Variable Importance in Projection (VIP) score was performed with the aim of identifying characteristic compounds for the investigated classes and performing feature selection, considering a threshold score of 1.5 [27]. The compounds selected as VIPs for both models were finally submitted to annotation. Annotations were manually reviewed combining HRMS with GC retention information, achieving identification confidence level 1 and level 2 according to the scale proposed by Schymanski et al. [28]. In particular, both the total score (> 80) and the ΔRI (ΔRI < 20) were considered. The total score combines several metrics including the SI (search index), RSI (reverse search index), HRF (high resolution filtering) and RHRF (reverse high-resolution filtering). In case of unavailability of the tabulated RI, the total score threshold was increased to 90.

GC × GC–MS chromatograms were processed using the Untargeted − Targeted (UT) fingerprinting approach [29], with the following processing parameters: signal-to-noise (S/N) threshold of 100 data points (dp) to include a peak or peak region into a template and a distance threshold of 10 dp as search space in the retention times domain in both ^1^D and ^2^D. Template matching was considered reliable with a Direct Match Factor (DMF) value of 700 or higher. Putative identification of the analytes was carried out by comparing the EI 70 eV spectra with those stored in the NIST 2023 database and the linear retention indices using a ± 20 dp tolerance in the ^1^D.

The feature template was created by including all the analysed samples with the “most relaxed” options, *i.e.*, considering a peak as “reliable” only if it was recognized in at least 50% + 1 of the chromatograms. The reliable peaks were then used as “anchor points” to automatically correct eventual ^1^D and ^2^D shifts throughout the analytical batch by applying polynomial second order transformations with a routine integrated in the data processing software. After the alignment step, a general “class image” that included the averaged signals of all the analysed samples was created to generate the feature template. This template includes both peaks and peak-regions that are used to create a consensus data matrix further used to compare the chromatograms.

## Results and discussion

### GC-Orbitrap HRMS results and data pre-processing

The complexity of HRMS data obtained from untargeted analysis requires a multi-stage data analysis workflow to extract meaningful information, namely for the purpose of this study the variables responsible for sample classification according to both material composition and intended end-use. Representative TIC chromatograms from GC-Orbitrap HRMS data are shown for one sample for each material are shown in Fig. S3. Applying the workflow devised for GC-HRMS data processing (Fig. S2), 73 features were filtered out after background subtraction (sample/blank area ratio ≥ 5), considering procedural blanks out of a total of 646 hits. For the remaining 573, additional filtration criteria based on analytical quality were implemented. Applying the data filtering strategy devised using “Max. Corrected QC Area RSD” and “Peak rating” parameters reduced the number of features to 459. Features ranging from C11 to over C28 with a wide range of polarity from linear alkanes to alcohols and esters were detected. The resulting dataset was submitted to multivariate statistical analysis to explore its structure and find the most significant features characteristic of the categories under investigation.

### Grouping of bioplastic samples based on indications of the maximum temperature for end-use and identification of their characteristic compounds

A first analysis of the dataset aimed to select the variables responsible for the sample clustering according to their low temperature (40 °C) or moderate temperature (70 °C) applications. As biobased plastics, such as PLA, often exhibit limited thermal stability, manufacturers overcome this drawback offering materials with a higher degree of crystallinity or blended formulations, enabling the production of cutlery capable of withstanding temperatures above 70 °C. However, these materials entail higher production costs, and labels lack clear details on their composition. Therefore, our work aims to develop an approach to discriminate samples based on both their composition and their degree of crystallinity. It is worth noting that PLA samples fall into the 40 °C category, whereas cPLA is intended for use at higher temperature (70 °C), along with no-PLA items. Indeed, the crystallisation process improves heat resistance and gas barrier properties of the material [30].

Explorative PCA analysis was performed on the filtered dataset: sample P10 which was identified as an outlier, resulting in high values for both T^2^ Hotelling and Q residuals, was therefore removed from the dataset. A new PCA was then performed on the clean dataset including 7 latent variables, accounting for 71.7% of the total variance. The resulting score plot showed patterns that did not differentiate the groups based on the measured variables in the first two principal components (Fig. S4).

Therefore, a supervised approach provided a reliable PLS-DA model including 2 latent variables. The model was externally validated using the test set that shows excellent performance in terms of sensitivity, specificity and precision (Table S2). VIP scores calculated on the PLS-DA model allowed to determine 52 key features (VIP score ≥ 1.5) responsible for classification.

Table 1 lists the selected compounds as (i) putatively identified, (ii) assigned to the hydrocarbon class (HC), (iii) associated as belonging to the same class (related compounds) based on common series of ions (Class), or (iv) indicated as unknown (U). In the last two cases, the compounds are missing from the mass databases and spectral libraries.

**Table 1:** List of compounds (VIPs) that discriminate bioplastics samples based on the indications of the maximum end-use temperature (40 °C, 70 °C). Categories are reported as follows: A – Hydrocarbons; B – Plasticisers; C – Oligomers; D – Other identified compounds including alkenes, lactones, carboxylic acids, alcohols, amides; E – Unknown

Code	Name	CAS number	Exact mass	Reference ion (m/z)	Mass error (ppm)^a^	RT (min)	RI_exp_^b^	ΔRI ^c^	Total score	HRF score	SI	Level of identification confidence^d^	Category
V1	Undecane	1120–21-4	156.1873	71.0855	−0.42	8.251	1100	0	95.7	99.7	792	1	A
V2	4-Methyl-1-undecene	74,630–39-0	168.1873	71.0855	−0.42	8.253	1104	19	95.6	100	780	2	D
V3	3-Methyl-1,2,4-cyclopentanetrione	4505–54-8	126.0312	83.01272	−0.36	8.400	1108	18	94.5	98.1	763	2	B
V4	3-Methyl-4-penten-2-ol	1569–59-1	100.0883	56.02563	0.00	8.610	1124	-	94.3	99.2	729	2	D
V5	U 1	-	-	124.08827	-	9.164	1154	-	-	-	-	5	E
V6	3,6-Dimethyl-1,4-dioxane-2,5-dione	95–96-5	144.0417	45.03345	0.89	9.934	1226	20	96.8	99.4	850	2	C
V7	U 2	-	-	70.07768	-	10.778	1245	-	-	-	-	5	E
V8	2-Methyldodecane	1560–97-0	184.2186	85.10116	−0.24	11.171	1267	3	94.9	99.3	758	2	A
V9	2,6,11-Trimethyldodecane	31,295–56-4	212.2499	85.10116	−0.24	11.270	1273	2	95.7	99.9	788	2	A
V10	2-Hexynyl aldehyde diethyl acetal	n.a	170.1301	140.08324	0.43	11.396	1280	-	93.6	99	701	2	D
V11	2-Butyl-1-octanol	3913–02–8	186.1978	85.10116	−0.24	11.42	1281	4	95.7	99.8	786	2	D
V12	HC 1	-	-	85.10116	−0.24	11.522	1287	-	-	-	-	3	A
V13	Tridecane	629–50-5	184.2186	85.10114	−0.47	11.714	1300	0	95.2	100	759	1	A
V14	3-Tridecene	41,446–53-1	182.2029	83.08549	2.59	11.716	1302	10	94.8	99.5	752	2	D
V15	2-Tridecene	41,446–59-7	182.2029	83.08549	2.59	11.864	1307	5	94.4	100	720	2	D
V16	4,6-Dimethyl-dodecane	61,141–72-8	198.2342	85.10116	−0.24	11.967	1313	12	94.6	99.6	737	2	A
V17	4-Methyl-2-decene	74,630–30-1	154.1716	85.10116	−0.24	12.005	1315	-	94.3	98.8	738	2	D
V18	2,3,5,8-Tetramethyldecane	192,823–15-7	198.2342	85.10116	−0.24	12.069	1319	1	95.4	98.1	808	2	A
V19	2-Ethylhexyl glycidyl ether	2461–15-6	186.1614	71.08553	0.00	12.299	1333	10	94	99.9	700	2	D
V20	HC 2	-	-	85.10116	−0.24	12.374	1338	-	-	-	-	3	A
V21	U 3	-	-	115.03888	-	12.581	1350	-	-	-	-	5	E
V22	Class A 1	-	-	117.05458	1.54	13.298	1393	-	-	-	-	3	C
V23	Dodecanal	112–54-9	184.1822	82.07764	−0.73	13.517	1406	3	94.2	96.4	784	2	D
V24	Tetradecane	629–59-4	198.2342	99.11682	−0.10	13.557	1400	9	94.5	98.8	749	1	A
V25	Class B 1	-	-	192.15089	-	13.912	1431	-	-	-	-	3	C
V26	Class A 2	-	-	129.05458	-	14.113	1443	-	-	-	-	3	C
V27	Class A 3	-	-	117.05459	1.62	14.263	1452	-	-	-	-	3	C
V28	3,4,5,6-Tetramethyloctane	62,185–21-1	170.2029	85.10116	−0.24	15.411	1525	-	94.5	98.4	759	2	A
V29	HC 3	-	-	71.0855	−0.42	15.473	1529	-	-	-	-	3	A
V30	HC 4	-	-	71.0855	−0.42	15.625	1540	-	-	-	-	3	A
V31	Class C 1	-	-	128.04677	−0.23	16.080	1571	-	-	-	-	3	C
V32	Class C 2	-	-	128.04677	−0.23	16.605	1607	-	-	-	-	3	C
V33	HC 5	-	-	113.13247	−0.09	16.846	1624	-	-	-	-	3	A
V34	Octylacetophenone	10,541–56-7	232.1822	232.18211	−0.43	17.225	1651	-	93.9	98.8	721	2	D
V35	HC 6	-	-	85.10114	−0.47	17.287	1655	-	-	-	-	3	A
V36	HC 7	-	-	71.08553	0.00	18.477	1741	-	-	-	-	3	A
V37	HC 8	-	-	85.10117	−0.12	18.662	1755	-	-	-	-	3	A
V38	HC 9	-	-	85.10117	−0.12	18.748	1761	-	-	-	-	3	A
V39	HC 10	-	-	85.10117	−0.12	20.163	1869	-	-	-	-	3	A
V40	Class C 3	-	-	200.06787	0.00	20.245	1875	-	-	-	-	3	C
V41	2-Phenyltridecane	4534–53-6	260.24985	85.10114	−0.47	20.658	1908	8	97.4	99.8	872	2	D
V42	HC 11	-	-	71.0855	−0.42	20.757	1916	-	-	-	-	3	A
V43	Class C 4	-	-	128.04672	−1.68	20.874	1925	-	-	-	-	3	C
V44	U 4	-	-	245.15358	-	20.894	1927	-	-	-	-	5	E
V45	Class C 5	-	-	128.04677	−0.23	21.101	1944	-	-	-	-	3	C
V46	2-Methylnonadecane	1560–86-7	282.3281	85.10118	0.00	21.404	1968	6	95.2	100	758	2	A
V47	5-Hydroxy-3,3,4,6,7-pentamethyl-1-benzofuran-2-one	86,563–00-0	220.1094	220.10942	0.14	21.850	2005	-	92.2	97.5	658	2	D
V48	HC 12	-	-	113.1325	0.08	22.344	2047	-	-	-	-	3	A
V49	U 5	-	-	114.05492	-	24.267	2217	-	-	-	-	5	E
V50	Class C 6	-	-	272.08954	2.02	24.424	2232	-	-	-	-	3	C
V51	Class C 7	-	-	200.06787	0.00	24.722	2260	-	-	-	-	3	C
V52	Class C 8	-	-	128.04677	−0.23	24.907	2277	-	-	-	-	3	C
V53	Class C 9	-	-	128.04677	−0.23	25.117	2293	-	-	-	-	3	C

^a^Mass error measured on the reference ion

^b^RI_exp_ experimental retention index

^c^ΔRI difference between experimental and tabulated retention indices

^d^Level of identification confidence achieved for each marker according to Schymanski et al. [28]. Level 1, confidently identified compounds, confirmed using reference standards; Level 2, putatively annotated compounds; Level 3, tentative candidates

Concerning identification of volatile compounds, it should be noted that the advantage of GC-HRMS lies in providing rich, multidimensional data, that is essential for the identification and structural annotation of the detected chemical features, including various fragmentation modes, accurate mass, retention information, and isotopic pattern. In particular, GC-Orbitrap HRMS with EI reduces ambiguities in compound identification by combining high-confidence structural fingerprints with accurate mass measurements, with the Orbitrap analyser capable of providing the exact mass with sub-ppm mass accuracy [31]. On the other hand, GC-Orbitrap produces complex datasets (full scan HRMS data) that require significant data storage and advanced, time-consuming processing workflows compared to the relatively quick analysis of conventional GC–MS. Its implementation in research laboratories entails high acquisition and maintenance costs and requires access to highly specialised personnel for data management and interpretation. Furthermore, the limited availability of complete, high-resolution spectral libraries compared to the extensive conventional unit-mass GC–MS databases can reduce the reliability of automatic library matching, often necessitating time-consuming manual validation or the implementation of home-made libraries.

Considering the VIPs responsible for classification based on the maximum end-use temperature, 50% consisted of hydrocarbons (either fully annotated and class-annotated), 27% were putatively identified compounds (aldehydes, ketones, furans, alkylphenyls, alcohols, alkenes), 17% were PLA oligomers and 6% were unknowns.

Among VIPs, several ion series with excellent mass accuracy were observed, deriving from related compounds with a similar chemical structure. In particular, three different ion series were observed (namely Class A, Class B and Class C), sharing the ion at *m/z* 56.02563. The other ions characterizing each Class are the following: *m/z* 73.06476, *m/z* 117.05458, *m/z* 128.08315, *m/z* 201.07568 for Class A; *m/z* 99.04404, *m/z* 114.06753, *m/z* 145.04955, *m/z* 219.17418 for Class B; *m/z* 128.04677, *m/z* 200.06787, *m/z* 272.08899 for Class C.

As no molecular ions were observed, only fragment ions and their associated neutral losses could be considered. Based on the detected ions and the corresponding MS fragmentation patterns, the neutral losses were determined, allowing us to formulate a reasonable hypothesis leading to the formation of the common ion [C_3_H_4_O]^+^ (Fig. S5).

Class C is the most represented (9 VIP compounds), with ions separated by 72.01112, which corresponds to the exact mass of the repeating unit of PLA (C_3_H_4_O_2_, mass error: 0.14 ppm). The characteristic EI spectrum of Class C features is reported in Fig. S6. The detected ions can be interpreted as fragments of cyclic PLA oligomers, as reported in the literature [12, 32]. The PLA oligomers assigned to Class C eluted within the RI range of 1607 and 2296. Along the chromatogram, they formed three distinct groups of peaks separated by approximately 280 RI units. Only two oligomers fall into the first group, whereas the second and third groups included three and four oligomers, respectively. This regularity in the chromatographic elution and the measured ΔRI supported the hypothesis that the three groups differ in terms of the number of repetition units, as previously observed by Veenaas and Haglund for PEG oligomers [33]. In addition, moving from the first to the third group, additional ions corresponding to an increase in the number of repeating unit (Δm: 72.02112 Da) were unequivocally detected thanks to the use of HRMS. For the oligomers of the third group (from Class C 6 to Class C 9 compounds), the ion at *m/z* 344.11011 was also observed in the deconvoluted mass spectra, although it was excluded by CD processing because its intensity was below the threshold set at 50,000. Within each group, the oligomers were separated by 17–50 RI units, suggesting they are likely isomers with the same number of repeating units.

As for the compounds belonging to Classes A and B, their characteristic ions have never been reported in the literature; however, a straightforward fragmentation pathway was observed yielding the common ion [CH_4_O]^+^ via neutral losses of C_4_H_8_ and C_3_H_6_O for Class A and Class B, respectively. Except for dodecanal, the only aldehyde identified in the studied samples, which has been previously reported as a NIAS odorant compound in PLA-based material [12, 34], most of the.

compounds were annotated as saturated hydrocarbons, belonging to the class of mineral oil saturated hydrocarbons (MOSH). Due to the presence of numerous structural isomers and the absence of a detectable molecular ion resulting from EI ionisation, a complete chemical characterization of the MOSH was not possible. Therefore, if no annotation proposal with ΔRI ≤ 10 was present, they were generically annotated as HC with a confidence level 3; conversely, linear hydrocarbons were annotated with a confidence level 1 due to their confirmation by standard injection for RI calculation.

MOSH can originate in manufacturing items from IAS like lubricants and external plasticisers, but also from NIAS deriving from the presence of other polymers in the material blend [35, 36], such as polyolefins. In particular, polyolefin oligomeric saturated hydrocarbons (POSH), that are branched hydrocarbons comparable to MOSH, can derive from material degradation [40]. Regulatory assessments highlight that toxicological concern is mainly associated with aromatic fractions, while the relevance of saturated hydrocarbons depends on their carbon‑number distribution and exposure scenario; therefore, their occurrence was discussed here primarily in terms of origin (IAS/NIAS) rather than hazard [38]. Depending on their pathway of formation, alkanes and alkenes may be identified either as NIAS, stemming from the thermo-oxidative degradation of other compounds, or as IAS, due to their frequent implementation as sustainable, bio-based plasticisers. Their use in the latter category is often justified by their non-toxicity and high biodegradability [39]. The presence of plasticisers is essential to reduce the fragility of thin bioplastic items such as disposable FCMs [40]. It is worth noting that some linear alkanes such as undecane, tridecane and tetradecane, and other compounds generically annotated as “alkane” have been previously detected in PLA samples [12].

**Fig. 1:**
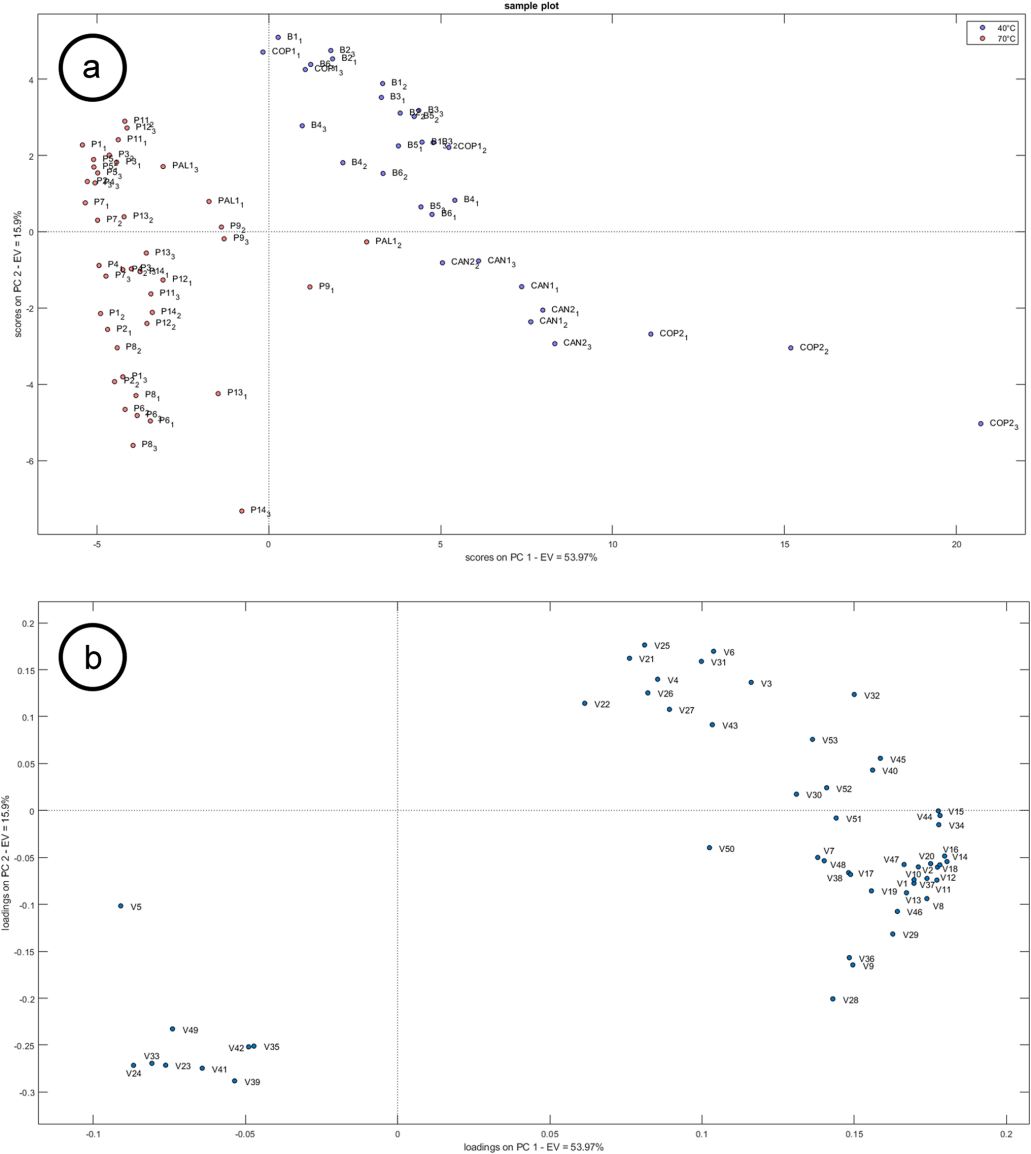
PCA analysis using VIPs as variables: (**a**) score plot (**b**) loading plot. Samples are labelled according to indications of the maximum temperature for end-use. Variables are reported in coded form according to Table 1

Based on the features extracted using the VIP scores, a new PCA analysis was carried out, labelling the scores according to the intended use of the bioplastic items. The first two PCs accounted for 69.87% of the total variance, with good clustering observed among the groups based on their intended use temperatures (Fig. 1a), with PC1 accounting for the primary separation. Figure 1b shows the loading plot: all PLA oligomers, as well as most of the annotated VIPs and hydrocarbons, exhibit a positive PC1 loading, thus characterising PLA items intended for end-use up to 40°C. This behaviour can be explained by the greater homogeneity of the products belonging to this group, which are all made of non-crystallized PLA polymer. On the other hand, the few features characterising items intended for end-use up to 70 °C are found at negative PC1 and PC2 values. This can be explained considering that the nature of the objects belonging to the higher temperature group (70 °C) was more heterogeneous since they were composed of cPLA and various unknown bioplastics reported as no-PLA, resulting in a lower number of key compounds. The different behaviour between PLA and cPLA items could be explained considering the effect of crystallinity on the polymer properties. At first, the crystalline portions of the material act as a barrier and inhibit swelling: additives and oligomers remain trapped in the crystalline regions, thus reducing their extractability with solvents or migration into food simulants [30, 41].

PLA oligomers are recognised as NIAS and can be by-products of polymer production or may result from degradation during material processing [42]. Due to the rigid crystalline regions, cPLA is more resistant to degradation, especially to hydrolysis process, than the amorphous material, thus producing low amounts of oligomers [43]. Another PLA-related compound, which characterizes non-crystalline PLA, is 3,6-dimethyl-1,4-dioxane-2,5-dione, a NIAS also known as lactide; it is the cyclic dimer of lactic acid, from which PLA can be obtained industrially through ring-opening polymerisation [44].

### Grouping of bioplastic samples based on the type of material resistant to moderate temperatures and identification of characteristics compounds

To further investigate the heterogeneous group of bioplastic items intended for end-use up to 70 °C, attention was focused on grouping these samples according to the material composition, i.e. cPLA *vs*. no-PLA. Again, since the exploratory PCA did not show significant clustering, a PLS-DA model was built including 2 latent variables. The classification performance of the PLS-DA model is reported in Table S2. Based on VIP scores, a total of 41 features were selected following the approach reported in the section “Untargeted workflow and multivariate analysis” (Table 2).

**Table 2:** List of compounds (VIPs) that discriminate bioplastics samples based on materials resistant to moderate temperatures (cPLA, no-PLA). Categories were reported as follows: A – Hydrocarbons; B – Plasticisers; C – Oligomers; D – Other identified compounds including alkenes, lactones, carboxylic acids, alcohols, amides; E – Unknown

Code	Name	CAS number	Exact mass	Reference ion (m/z)	Mass error (ppm)^a^	RT (min)	RI_exp_^b^	ΔRI ^c^	Total score	HRF score	SI	Level of identification confidence^d^	Category
V54	LA ester 1	-	-	88.05187	-	8.447	1115	-	-	-	-	3	C
V55	U 6	-	-	227.03972	-	9.087	1150	-	-	-	-	5	E
V5	U 1^e^	-	-	124.08827	-	9.164	1154	-	-	-	-	5	E
V56	Diethyl ketomalonate	609–09-6	174.0523	102.06752	0.61	9.634	1180	-	94.3	99.6	721	2	B
V57	Class D 1	-	-	130.02608	0.15	10.623	1236	-	-	-	-	3	C
V58	Lactic acid	50–21-5	90.03115	89.05971	−0.34	10.747	1243	-	92.6	95.9	709	2	C
V59	1-Decanol	112–30-1	158.1665	56.06204	−0.18	11.202	1269	3	96.9	99.8	848	2	D
V60	Class D 2	-	-	130.02606	0.00	11.358	1278	-	-	-	-	3	C
V61	4-tert-Butylphenol	98–54-4	150.1039	150.10388	0.13	11.544	1288	7	88.1	85.2	700	2	D
V19	2-Ethylhexyl glycidyl ether^e^	2461–15-6	186.1614	71.08553	0.00	12.299	1333	10	94	99.9	700	2	B
V62	Dibutyl succinate	141–03–7	230.1513	101.02341	3.59	12.44	1342	-	93.7	99.8	688	2	C
V63	U 7	-	-	81.03347	-	12.855	1367	-	-	-	-	5	E
V64	U 8	-	-	68.02567	-	12.855	1367	-	-	-	-	5	E
V65	Class D 3	-	-	99.04406	-	12.879	1368	-	-	-	-	3	C
V24	Tetradecane^e^	629–59-4	198.2342	99.11682	−0.10	13.557	1400	0	94.5	98.8	749	1	A
V66	Dimethyl methylglutarate	14,035–94-0	174.0887	115.07527	−0.78	13.835	1426	-	93.4	99.4	680	2	B
V67	Dimethyl phthalate	131–11-3	194.0574	92.06271	0.55	14.131	1444	4	98.1	100	906	2	B
V68	Octylcyclohexane	1795–15-9	196.2186	56.06203	0.79	14.19	1448	0	92.9	99.8	647	2	A
V69	LA ester 2	-	-	89.05973	-	15.362	1522	-	-	-	-	3	C
V70	U 9	-	-	58.07323	-	15.409	1525	-	-	-	-	5	E
V71	1,6-Dioxacyclododecane-7,12-dione	777–95-7	200.1043	84.05695	0.24	15.687	1544	-	95.6	99.9	780	2	B
V72	U 10	-	-	114.06753	-	15.923	1560	-	-	-	-	5	E
V73	U 11	-	-	85.06477	-	16.548	1603	-	-	-	-	5	E
V74	HC 13	-	-	85.10117	−0.12	16.561	1604	-	-	-	-	3	A
V32	Class C 2^e^	-	-	128.04677	−0.23	16.605	1607	-	-	-	-	3	C
V75	Butyl isobutyl glutarate	n.a	244.1669	115.0390	2.60	17.263	1653	6	91.9	99.6	602	2	B
V76	1,3-Diphenylpropane	1081–75-0	196.1247	93.06538	−3.17	17.272	1653	20	97.3	99.5	871	2	D
V77	Heptadecane	629–78-7	240.2812	43.05419	−0.86	17.89	1700	0	96.5	99.8	827	1	A
V78	1,2-Diphenylcyclopropane	29,881–14-9	194.1090	179.0855	−0.15	18.422	1737	-	95	96.4	821	2	D
V79	U 12	-	-	112.1245	-	18.645	1753	-	-	-	-	5	E
V80	HC 9	-	-	85.10117	−0.12	19.777	1839	-	-	-	-	3	A
V81	Class E 1	-	-	145.0496	-	20.026	1858	-	-	-	-	3	C
V82	HC 10	-	-	85.10117	−0.12	20.163	1869	-	-	-	-	3	A
V83	Nonadecane	629–92-5	268.3125	99.11682	−0.10	20.653	1900	0	95.4	99.8	772	1	A
V84	2-Phenyltridecane	4534–53-6	260.2499	85.10114	−0.47	20.658	1908	8	97.4	99.8	872	1	A
V85	1,2-Epoxyoctadecane	7390–81-0	268.2761	82.07767	−0.36	20.753	1915	15	92.4	98.4	649	2	D
V86	Ethyl stearate	111–61-5	312.3023	88.05187	−0.11	23.958	2189	6	88.2	99.8	415	2	B
V49	U 5^e^	-	-	114.0549	-	24.267	2217	-	-	-	-	5	E
V87	1,2-Cyclohexanedicarboxylic acid, decyl isobutyl ester	n.a	368.2921	126.06752	0.08	26.916	2458	19	92.8	99.6	649	2	B
V88	Succinic acid ester	-	-	71.04913	-	27.543	2538	-	-	-	-	3	C
V89	1,2-Cyclohexanedicarboxylic acid, 3-methylphenyl nonyl ester	n.a	388.2608	80.06203	−0.25	30.171	2821	5	93.2	99.2	675	2	B

^a^Mass error measured on the reference ion

^b^RI_exp_ experimental retention index

^c^ΔRI difference between experimental and tabulated retention indices

^d^Level of identification confidence achieved for each marker according to Schymanski et al. [28]. Level 1, confidently identified compounds, confirmed using reference standards; Level 2, putatively annotated compounds; Level 3, tentative candidates

^e^Features in common with VIPs calculated for the model based on the indications of the maximum temperature for end-use

Among the selected VIP features, 14 putatively identified compounds, 8 HC, 8 PLA-related compounds (lactic acid, lactate esters, and PLA oligomers), 2 PBS-related compounds, and 9 unknowns were observed. In particular, compounds belonging to hydrocarbons and esters were also annotated in a previously published article [24].

Class D is characterised by the *m/z* 56.02563, *m/z* 102.03114, and *m/z* 130.02606 ions, with the former in common with Classes A, B and C (PLA-related compounds). In this case, a two-step rearrangement was hypothesized, with the loss of 27.99492 Da and 46.00552 Da, related to CO and CH_2_O_2_ respectively (Fig. S5). The spectrum of the compound belonging to Class E is characterised by two different ion series. The first, showing lower abundance, matched the ions belonging to Class C, while a more abundant series presented *m/z* values shifted by 17.0028 units from that of Class C compounds (*m/z*: 73.02839, 145.04958, 217.0706). The observed shift can be explained by the presence of a hydroxyl group (mass error: −0.59 ppm), suggesting that the molecule is a hydrolysed open-chain PLA oligomer in accordance with the previous literature annotation of the same ion pattern [45].

**Fig. 2:**
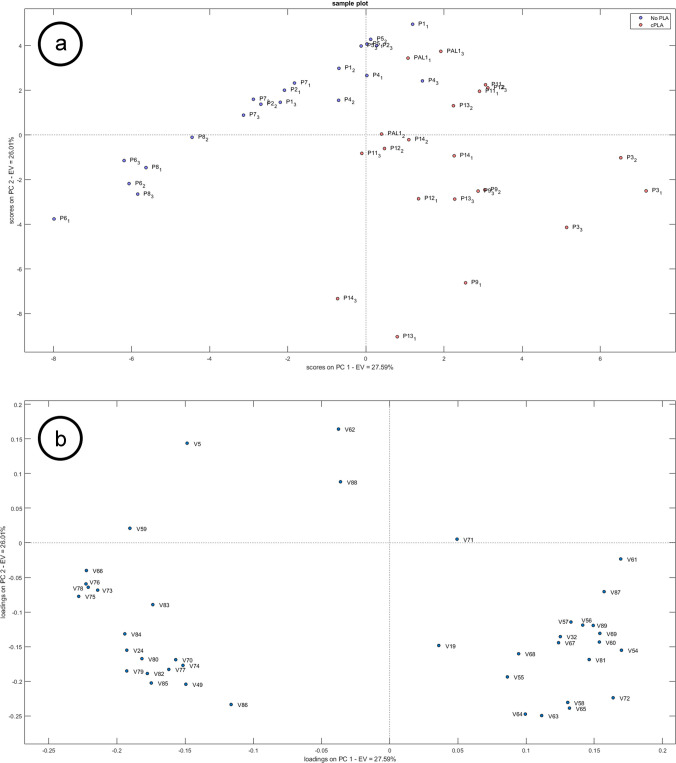
PCA using VIPs as variables: (**a**) score plot, (**b**) loading plot. Samples are labelled according to the type of material resistant to moderate temperatures (cPLA, no-PLA). Variables are reported in coded form according to Table 2

Partial clustering was observed when PCA was carried out on the selected VIP compounds (Fig. 2a) with the first two PCs accounting for 53.60% of the total variance.

As expected, cPLA samples were characterised predominantly by PLA-related VIPs (Fig. 2b), consisting of NIAS, namely lactic acid, lactic acid esters, and PLA oligomers, according to their main polymeric component. Generally, a lower number of hydrocarbons was observed compared to no-PLA samples [40].

Esters were found in all samples, since several lactates, succinates, citrates, phthalates are intentionally added substances commonly used as monomers or plasticisers [46, 47]. Some esters can be also considered NIAS as can originate from transesterification processes during material manufacturing [40]. In particular, cPLA samples were characterised by the presence of dicarboxylic esters, whereas no-PLA by single-function esters.

The two succinic acid esters, namely dibutyl succinate and an unidentified succinic acid ester, are present in both cPLA and no-PLA samples. They are recognised as NIAS that can be associated with the presence of PBS either as the blend polymer for cPLA samples [48], or as the main biopolymer in the no-PLA samples [49]. 1,6-Dioxacyclododecane-7,12-dione is a cyclic butylene adipate ester obtained as a by-product during the synthesis of polybutylene adipate (PBAT) and can be considered a NIAS [50]. It has been previously detected in various bioplastic-based FCMs by Wongphan et al. [51], suggesting the presence of PBAT in the formulation of cPLA samples. However, its presence could be also ascribed to cross-contamination during the manufacturing process.

### Optimisation of GC × GC–MS chromatographic conditions

In parallel with GC-HRMS analysis, the extracts were also analysed by GC × GC–MS. GC × GC offers a substantial advantage over ^1^D-GC because it combines two columns with different selectivity, increasing peak capacity and separation efficiency. The dual-column setup reduces co-elution and provides clearer resolution of complex mixtures, enabling a more detailed chemical characterisation. The added dimensionality also enhances pattern recognition, which reveals hidden correlations between compounds with structural or functional similarities, allowing to improve data interpretation by reducing noise and focusing on significant features [52, 53]. Preliminary experiments were carried out using different column combinations to find the optimal chromatographic separation conditions. In particular, the following combinations were tested on a pilot sample:

- a) ^1^D column Rtx-Wax; 30 m × 0.25 mm d_c_ × 0.50 μm d_f_; ^2^D column Rxi-1 MS; 1.0 m × 0.1 mm d_c_ × 0.53 μm d_f_
- b) ^1^D column Rxi-5Sil MS; 30 m × 0.25 mm d_c_ × 0.50 μm d_f_); ^2^D column Rxi-1 MS; 1.0 m × 0.1 mm d_c_ × 0.53 μm d_f_
- c) ^1^D column Rxi-5Sil MS; 30 m × 0.25 mm d_c_ × 0.50 μm d_f_); ^2^D column Rxi-17Sil MS; 1.0 m × 0.1 mm d_c_ × 0.10 μm d_f_

**Fig. 3:**
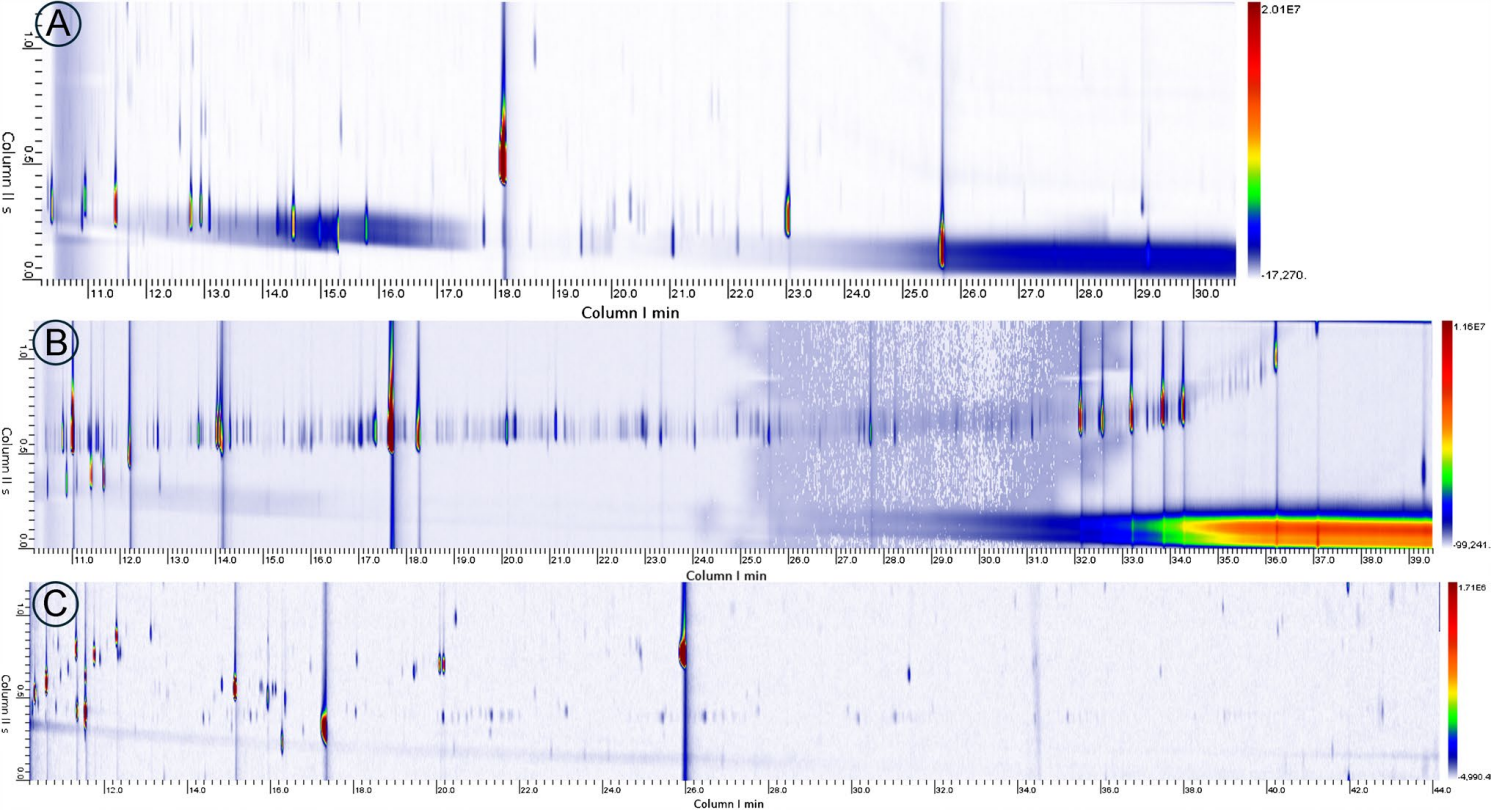
GC × GC chromatograms of PLA sample extract recorded using the following column combinations: (**a**) Rtx-Wax (30m, 0.25mm, 0.50μm) × Rxi-1 MS (1.0m, 0.1mm, 0.53μm); (**b**) Rxi-5Sil MS (30m, 0.25mm, 0.50μm) × Rxi-1 MS (1.0m, 0.1mm, 0.53μm); (**c**) Rxi-5Sil MS (30m, 0.25mm, 0.50μm) × Rxi-17Sil MS (1.0m, 0.1mm, 0.10μm)

The chromatograms of the three samples investigated obtained using each column combination are reported in Fig. 3.

The best results were obtained using column combination c. This is because this combination provides good orthogonality as the first, moderately polar column is complementary to the second, more polar column, resulting in greater peak dispersion in two-dimensional space and minimising co-elution. In contrast, column combination a, although apparently complementary, exhibits a large polarity gap, causing many compounds to cluster along the same retention regions in the second dimension. Furthermore, the Wax column imposes a lower maximum operating temperature, which limits the elution of high-boiling compounds and compresses late-eluting peaks, further reducing resolution for complex mixtures. Finally, the combination b does not exhibit a sufficient polarity difference between the two dimensions, resulting in overlap in complex regions. Overall, the Rxi-5Sil/Rxi-17Sil pairing demonstrates superior performance, allowing for clearer visualisation of chemical classes and more reliable identification of individual analytes. Wrap-around was observed with the more abundant peaks, but P_M_ was selected as a compromise to prioritise sensitivity in the diverting flow‑modulation set‑up while maintaining adequate sampling of the 1D peaks (≥ 3 slices per peak; MR ≥ 3).

### Integration of GC × GC–MS in enhancing GC-HRMS data interpretation

**Fig. 4:**
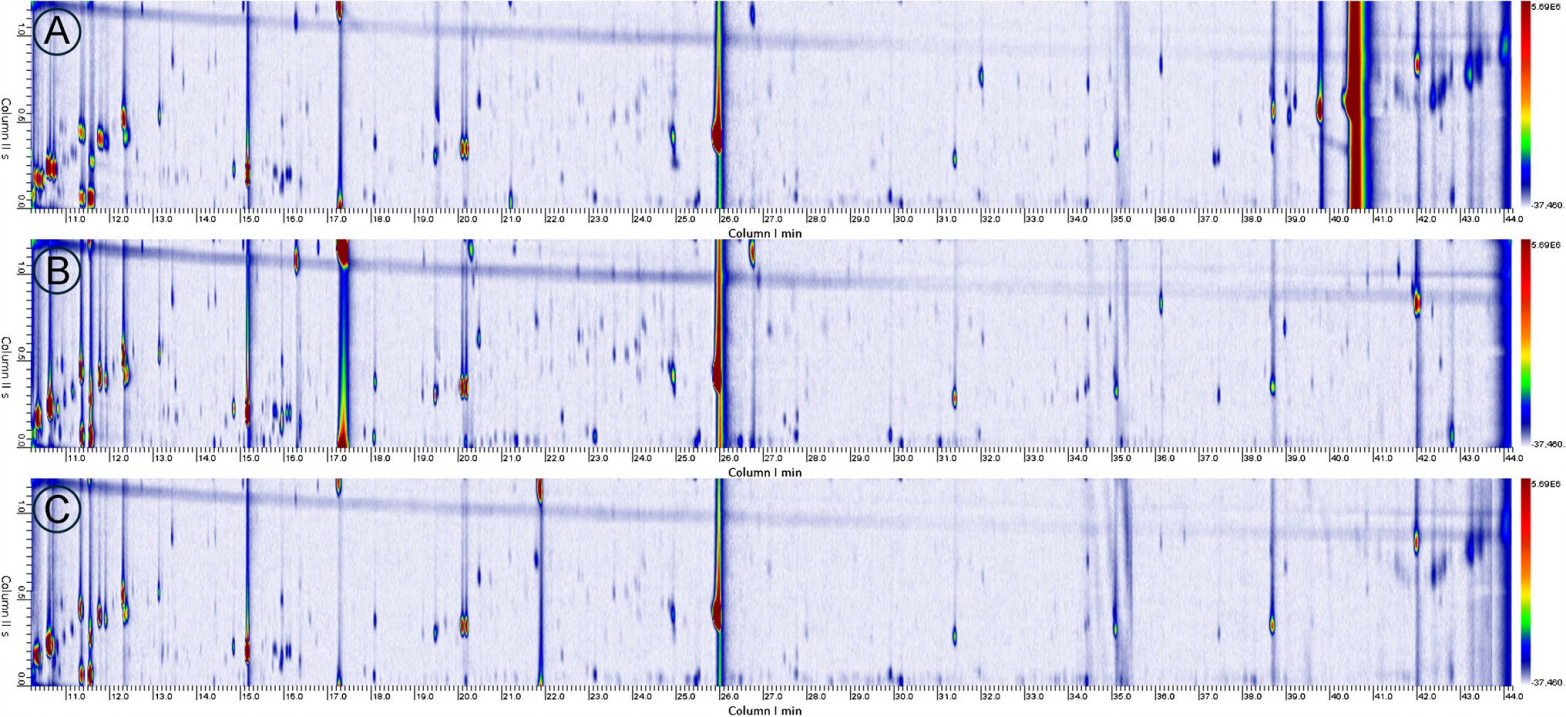
GC × GC–MS Class Images of cPLA (**a**), PLA (**b**), and no-PLA (**c**) samples

Given that the GC × GC–MS results aligned with GC-Orbitrap HRMS, demonstrating both methods reached the same high-level outcome, emphasis is placed on the specific analytical improvements provided by GC × GC–MS. As is well known, GC × GC–MS offers superior separation power, which significantly helps resolve co-eluting compounds that a 1D-GC system (even with HRMS) might miss, and provides structured chromatograms that aid in refining structural hypotheses [54]. In this work, GC × GC–MS was able to identify several compounds that had been excluded from the GC-Orbitrap HRMS dataset following the application of CD filters due to poor peak shape. The GC × GC–MS chromatograms of the PLA, cPLA and no-PLA class images are illustrated in Fig. 4.

A total of 31 analytes discriminating the bioplastic samples according to their material type were detected, 64% of which consisted of hydrocarbons, the majority being characteristic of the PLA class. Good complementary results were achieved by coupling GC × GC–MS with GC-Orbitrap HRMS.

Of the VIP compounds annotated by GC-Orbitrap HRMS as HC, GC × GC–MS was able to putatively identify HC6 and HC10 with a confidence level of 2, namely 4-methylhexadecane- (Exp. ^1^D-RI:1658) and 2-methyloctadecane (Exp. ^1^D-RI:1857).

Additionally, the averaged GC × GC-MSchromatograms for PLA, cPLA and no-PLA highlighted intense signals characterizing cPLA items (an example of the correspondent extracted ion chromatogram (XIC) of *m/z*: 259.20 is reported in Fig. S7. These peaks, partially retained in the second dimension, could be assigned to citric acid esters, including tributyl citrate (Exp. ^1^D-RI: 2189) and acetyl tributyl citrate (Exp. ^1^D-RI: 2266). The presence of citric acid esters in CPME extracts of bioplastic materials has been reported in our previous study [24]. Citrates belong to a widely recognised class of plasticisers (IAS) representing the standard for manufacturing food packaging, children’s toys and medical devices [55], despite the relative high costs. In fact, these compounds are considered environmentally friendly, safe, non-toxic, and able to prevent precipitation [56]. Therefore, they are widely used to enhance flexibility, processability, and impact resistance in rigid polymers like cPLA [37]. Among these, triethyl citrate and tributyl citrate, along with their acetylated derivatives exhibit distinct performance profiles. Ethyl citrate esters, which are characterized by a lower molecular weight and higher polarity, provide an efficient reduction of the glass transition temperature and promote crystallization at relatively low concentrations; however, their higher volatility and migration tendency limit their suitability for long-term applications. In contrast, butyl citrate derivatives possess bulkier alkyl chains, resulting in lower volatility, improved permanence, and enhanced mechanical toughness, making them preferable for PLA formulations intended for food contact and medical use. In the analysed samples, only butyl citrate derivatives were detected in cPLA samples (characteristic *m/z* fragmentation pattern: 259, 185, 129, 57), while no ethyl citrate-related pattern was detected (characteristic *m/z* fragmentation pattern: 203, 157, 115).

**Fig. 5:**
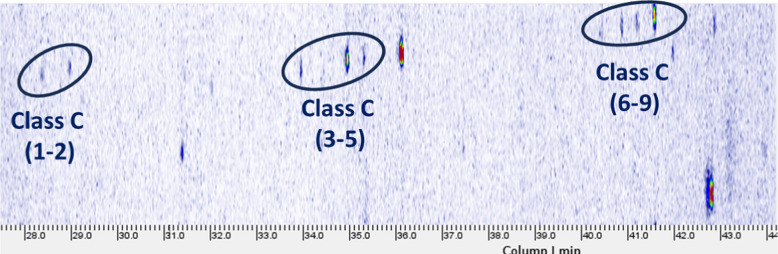
GC × GC–MS extracted ion chromatogram (XIC) of m/z 128.04 in a PLA sample extract

Regarding cyclic PLA oligomers, which belong to the NIAS class and were extensively characterised in the GC‑Orbitrap HRMS dataset, GC × GC–MS analysis offered a complementary insight that helped refine their interpretation. Although none of the compounds assigned to Classes A and B were detected in the GC × GC chromatograms, likely due to their low concentrations, Class C oligomers showed an interesting pattern on the 2D chromatographic plane. In the two‑dimensional XIC chromatogram (*m/z*: 128.04), three separate groups of analytes could be distinguished (Fig. 5).

These groups showed a consistent pattern in which oligomers eluting later in the first dimension were also more highly retained in the second, reflecting differences in both molecular size and polarity between species with increasing numbers of repeating units. Since the 2D column was operated at constant flow, the correlation between later 1D elution and higher apparent 2D retention is not explained by changes in 2D flow during the run; nonetheless, 2D retention remains method-dependent, and the trend is therefore interpreted qualitatively. The 1D column spreads the oligomers according to differences in volatility, while the 2D one adds an orthogonal contribution, responding to the ester‑rich character of these molecules. Although cyclic PLA oligomers are not highly polar, their multiple ester groups introduce enough interaction with the secondary phase to produce measurable differences in retention. As a result, oligomers with additional repeating units not only appear later in 1D but also shift upward in 2D, giving rise to the three distinct clusters observed in the GC × GC–MS plane. The distribution of these peaks across the GC × GC plane agrees with the oligomer series identified in the HRMS data, further supporting their assignment as cyclic PLA oligomers differing by increments of the PLA repeating unit (Δ*m/z*: ≈ 72).

## Conclusions and outlook

This work highlights the importance of detecting the presence of IAS and NIAS and identifying related molecules, not only for safety assessment purposes but also for gathering information on the source of the material used to produce a biobased product. Thanks to the advanced techniques and combined approach used, the results demonstrated that it is possible to obtain information on data derived from low levels of additives or impurities present in a material to understand its origin. Furthermore, data processing made it possible for the first time to discriminate between samples based on both material composition and maximum end-use temperature. The two platforms used here, GC × GC-TOF–MS and GC-Orbitrap HRMS, complement each other by offering different strengths in chromatographic resolution and mass accuracy to cover a wide range of IAS and NIAS from PLA and no-PLA FCMs, enabling the detection of different compounds.

The application of supervised PLS-DA analysis to accurate GC-Orbitrap HRMS mass measurements enabled the reliable identification of key markers to differentiate between samples based on the type of bio-based material. IAS and NIAS like cyclic oligomers and hydrocarbons were found to characterise samples based on the maximum end-use temperature, while PLA-related molecules (lactic acid, lactate esters, and linear oligomers) and some hydrocarbons were found as key variables of the cPLA and no-PLA class, respectively when considering classification by the type of material resistant to moderate temperature. The GC × GC-TOF–MS analysis successfully identified distinct series of compounds, including PLA oligomers, citric acid esters as plasticisers, and mineral oil saturated hydrocarbons. The consistent, regular chromatographic patterns observed provided strong support for the structural hypotheses derived from the complementary GC–Orbitrap HRMS data. This integrated approach increased confidence in the characterisation of the complex mixture components, providing information that is relevant especially for safety assessment studies, where a toxicity-related investigation of identified substances is strongly encouraged.

## Supplementary Information

Below is the link to the electronic supplementary material.

Supplementary file1 (DOCX 1.80 MB)

## Data Availability

The datasets can be made available upon request.

## References

[1] Nanda S, Patra BR, Patel R, Bakos J, Dalai AK. Innovations in applications and prospects of bioplastics and biopolymers: a review. Environ Chem Lett. 2022;20:379–95. 10.1007/s10311-021-01334-4. 34867134 10.1007/s10311-021-01334-4 PMC8629338

[2] European Parliament and Council. Directive (EU) 2019/904 of 5 June 2019 on the reduction of the impact of certain plastic products on the environment. Off J Eur Union. 2019;L 155:1–19.

[3] Jariyasakoolroj P, Leelaphiwat P, Harnkarnsujarit N. Advances in research and development of bioplastic for food packaging. J Sci Food Agric. 2020;100:5032–45. 10.1002/jsfa.9497. 30450696 10.1002/jsfa.9497

[4] Mattarozzi M, Milioli M, Cavalieri C, Bianchi F, Careri M. Rapid desorption electrospray ionization-high resolution mass spectrometry method for the analysis of melamine migration from melamine tableware. Talanta. 2012;101:453–9. 10.1016/j.talanta.2012.09.059. 23158348 10.1016/j.talanta.2012.09.059

[5] Zimmermann L, Dombrowski A, Völker C, Wagner M. Are bioplastics and plant-based materials safer than conventional plastics? In vitro toxicity and chemical composition. Environ Int. 2020;145:106066. 10.1016/j.envint.2020.106066. 32951901 10.1016/j.envint.2020.106066

[6] Cruz RMS, Krauter V, Krauter S, Agriopoulou S, Weinrich R, Herbes C, et al. Bioplastics for food packaging: environmental impact, trends and regulatory aspects. Foods. 2022;11:3087. 10.3390/foods11193087. 36230164 10.3390/foods11193087 PMC9563026

[7] Riboni N, Bianchi F, Cavazza A, Piergiovanni M, Mattarozzi M, Careri M. Mass spectrometry-based techniques for the detection of non-intentionally added substances in bioplastics. Separations. 2023;10:222. 10.3390/separations10040222.

[8] Cavazza A, Mattarozzi M, Franzoni A, Careri M. A spotlight on analytical prospects in food allergens: from emerging allergens and novel foods to bioplastics and plant-based sustainable food contact materials. Food Chem. 2022;388:132951. 10.1016/j.foodchem.2022.132951. 35447585 10.1016/j.foodchem.2022.132951

[9] Piergiovanni M, Termopoli V, Maffezzoni C, Riboni N, Consonni V, Bianchi F, et al. Condensed phase membrane introduction mass spectrometry: a new frontier for the real-time monitoring of hazardous chemical migration from food contact materials. Green Anal Chem. 2025;12:100199. 10.1016/j.greeac.2024.100199.

[10] Sapozhnikova Y, Nuñez A, Johnston J. Screening of chemicals migrating from plastic food contact materials for oven and microwave applications by liquid and gas chromatography - Orbitrap mass spectrometry. J Chromatogr A. 2021;1651:462261. 10.1016/j.chroma.2021.462261. 34126375 10.1016/j.chroma.2021.462261

[11] Lin J, Wu WL, Zhong AH, Xian YP, Zhong HN, Dong B, et al. Non-targeted analysis and risk assessment of intentionally and non-intentionally added substances migrating from the emerging biodegradable food contact material poly(butylene adipate-co-terephthalate)/modified starch blend film. Food Packag Shelf Life. 2023;40:101190. 10.1016/j.fpsl.2023.101190.

[12] Vázquez-Loureiro P, Cariou R, Dervilly G, Le Bizec B, Lestido-Cardama A, Barbosa-Pereira L, et al. Identification of volatile and semi-volatile components in food contact bioplastics based on GC–MS non-targeted screening. J Chromatogr A. 2025;1762:466377. 10.1016/j.chroma.2025.466377. 40974706 10.1016/j.chroma.2025.466377

[13] Spinei M, Kurek M, Wrona M, Vandekinderen I, De B, Paseiro-Losada P. Identification and quantification of intentionally and non-intentionally added substances in food contact materials: a review of migration mechanisms and influencing factors. Food Res Int. 2025;183:116880. 10.1016/j.foodres.2025.116880. 10.1016/j.foodres.2025.116880 40790681

[14] Biedermann M, Grob K. Advantages of comprehensive two-dimensional gas chromatography for comprehensive analysis of potential migrants from food contact materials. Anal Chim Acta. 2019;1057:11–7. 10.1016/j.aca.2018.10.046. 30832909 10.1016/j.aca.2018.10.046

[15] Carrero-Carralero C, Escobar-Arnanz J, Ros M, Jiménez-Falcao S, Sanz ML, Ramos L. An untargeted evaluation of the volatile and semi-volatile compounds migrating into food simulants from polypropylene food containers by comprehensive two-dimensional gas chromatography-time-of-flight mass spectrometry. Talanta. 2019;195:800–6. 10.1016/j.talanta.2018.12.011. 30625621 10.1016/j.talanta.2018.12.011

[16] Hochegger A, Pantò S, Jones N, Leitner E. One-dimensional and comprehensive two-dimensional gas chromatographic approaches for the characterization of post-consumer recycled plastic materials. Anal Bioanal Chem. 2023;415:2447–57. 10.1007/s00216-023-04599-6. 36820911 10.1007/s00216-023-04599-6 PMC10149440

[17] Wolf N, Säger S, Lommatzsch M, Simat TJ. Analysis of volatile oxidized oligomers from polyolefins by off-line normal phase high performance liquid chromatography and one-dimensional and comprehensive two-dimensional gas chromatography. Polym Degrad Stab. 2021;185:109490. 10.1016/j.polymdegradstab.2021.109490.

[18] Omer E, Bichon E, Hutinet S, Royer A-L, Monteau F, Germon H, et al. Toward the characterisation of non-intentionally added substances migrating from polyester-polyurethane lacquers by comprehensive gas chromatography-mass spectrometry technologies. J Chromatogr A. 2019;1601:327–34. 10.1016/j.chroma.2019.05.024. 31128881 10.1016/j.chroma.2019.05.024

[19] Nerín C, Bourdoux S, Faust B, Gude T, Lesueur C, Simat T, et al. Guidance in selecting analytical techniques for identification and quantification of non-intentionally added substances (NIAS) in food contact materials (FCMS). Food Addit Contam. 2022;39:620–43. 10.1080/19440049.2021.2012599. 10.1080/19440049.2021.2012599 35081016

[20] Yusà V, López A, Dualde P, Pardo O, Fochi I, Miralles P, et al. Identification of 24 unknown substances (NIAS/IAS) from food contact polycarbonate by LC-Orbitrap Tribrid HRMS-DDMS3: safety assessment. Int J Anal Chem. 2021;2021:6654611. 10.1155/2021/6654611.

[21] Sapozhnikova Y. Non-targeted screening of chemicals migrating from paper-based food packaging by GC-Orbitrap mass spectrometry. Talanta. 2021;226:122120. 10.1016/j.talanta.2021.122120. 33676675 10.1016/j.talanta.2021.122120

[22] Belarbi S, Vivier M, Zaghouani W, De Sloovere A, Agasse-Peulon V, Cardinael P. Comparison of new approach of GC-HRMS (Q-Orbitrap) to GC–MS/MS (triple-quadrupole) in analyzing the pesticide residues and contaminants in complex food matrices. Food Chem. 2021;359:129932. 10.1016/j.foodchem.2021.129932. 33945988 10.1016/j.foodchem.2021.129932

[23] Miralles P, Fuentes-Ferragud E, Socas-Hernández C, Coscollà C. Recent trends and challenges on the non-targeted analysis and risk assessment of migrant non-intentionally added substances from plastic food contact materials. Toxics. 2025;13:543. 10.3390/toxics13070543. 40710987 10.3390/toxics13070543 PMC12299502

[24] Piergiovanni M, Fontanarosa M, Riboni N, Cavazza A, Mattarozzi M, Bianchi F, Careri M. A green ultrasound-assisted extraction method coupled to gas chromatography-mass spectrometry for the identification of intentionally and non-intentionally added substances in bio-based food contact materials. Green Anal. Chem. 2026;100330. 10.1016/j.greeac.2026.100330 10.1007/s00216-026-06444-y PMC13144257 41888411

[25] Ballabio D. A MATLAB toolbox for principal component analysis and unsupervised exploration of data structure. Chemom Intell Lab Syst. 2015;149:1–9. 10.1016/j.chemolab.2015.10.003.

[26] Ballabio D, Consonni V. Classification tools in chemistry. Part 1: linear models. PLS-DA. Anal Methods. 2013;5:3790–8. 10.1039/C3AY40582F.

[27] Chong IG, Jun CH. Performance of some variable selection methods when multicollinearity is present. Chemom Intell Lab Syst. 2005;78:103–12. 10.1016/j.chemolab.2004.12.011.

[28] Schymanski EL, Jeon J, Gulde R, Fenner K, Ruff M, Singer HP, et al. Identifying small molecules via high resolution mass spectrometry: communicating confidence. Environ Sci Technol. 2014;48(4):2097–8. 10.1021/es5002105. 24476540 10.1021/es5002105

[29] Stilo F, Liberto E, Spigolon N, Genova G, Rosso G, Fontana M, et al. An effective chromatographic fingerprinting workflow based on comprehensive two-dimensional gas chromatography – mass spectrometry to establish volatiles patterns discriminative of spoiled hazelnuts (*Corylus avellana* L.). Food Chem. 2021;340:128135. 10.1016/j.foodchem.2020.128135. 33011466 10.1016/j.foodchem.2020.128135

[30] Petrovics N, Kirchkeszner C, Patkó A, Tábi T, Magyar N, Kovácsné Székely I, et al. Effect of crystallinity on the migration of plastic additives from polylactic acid-based food contact plastics. Food Packag Shelf Life. 2023;36:101054. 10.1016/j.fpsl.2023.101054.

[31] Piergiovanni M, Giliberti C, Maffezzoni C, Errico D, Blandino M, Dall’Asta C, et al. Electronic nose technology for the detection of ergot al-kaloid in soft wheat and identification of the relevant volatile compounds by solid phase micro-extraction/gas chromatography-high resolution Orbitrap-mass spectrometry coupled to chemometrics. Food Chem. 2025;484:144455. 10.1016/j.foodchem.2025.144455. 40288212 10.1016/j.foodchem.2025.144455

[32] Kopinke FD, Remmler M, Mackenzie K. Thermal decomposition of biodegradable polyesters—II. Poly(lactic acid). Polym Degrad Stab. 1996;53(3):329–42. 10.1016/0141-3910(96)00102-4.

[33] Veenaas C, Haglund P. Retention-time prediction in comprehensive two-dimensional gas chromatography to aid identification of unknown contaminants. Anal Bioanal Chem. 2018;410(13):3047–59. 10.1007/s00216-018-1415-x. 30361914 10.1007/s00216-018-1415-x PMC6244764

[34] Ubeda S, Aznar M, Nerín C. Determination of volatile compounds and their sensory impact in a biopolymer based on polylactic acid (PLA) and polyester. Food Chem. 2019;294:171–8. 10.1016/j.foodchem.2019.05.069. 31126449 10.1016/j.foodchem.2019.05.069

[35] Mazidi MM, Arezoumand S, Zare L. Research progress in fully biorenewable tough blends of polylactide and green plasticizers. Int J Biol Macromol. 2024;279:135345. 10.1016/j.ijbiomac.2024.135345. 39244110 10.1016/j.ijbiomac.2024.135345

[36] Olonisakin K, Mohanty AK, Thimmanagari M, Misra M. Recent advances in biodegradable polymer blends and their biocomposites: a comprehensive review. Green Chem. 2025;27(27):5965–75. 10.1039/D5GC01294E.

[37] Biedermann-Brem S, Kasprick N, Simat T, Grob K. Migration of polyolefin oligomeric saturated hydrocarbons (POSH) into food. Food Addit Contam. 2012;29(3):1–12. 10.1080/19440049.2011.641164. 10.1080/19440049.2011.641164 22243490

[38] EFSA Panel on Contaminants in the Food Chain (CONTAM), Schrenk D, Bignami M, Bodin L, del Mazo J, Grasl‐Kraupp B, Hogstrand C, Hoogenboom L (Ron), Leblanc J, Nebbia CS, Nielsen E, Ntzani E, Petersen A, Sand S, Schwerdtle T, Vleminckx C, Wallace H, Alexander J, Goldbeck C, Grob K, Gómez Ruiz JÁ, Mosbach‐Schulz O, Binaglia M, Chipman JK. Update of the risk assessment of mineral oil hydrocarbons in food. EFS2. 2023;21:9. 10.2903/j.efsa.2023.8215 10.2903/j.efsa.2023.8215 PMC10498375 37711880

[39] Paiva R, Wrona M, Nerín C, et al. Volatile compounds and off-odors analysis of recycled PLA for packaging applications: an essential factor for ensuring food safety and quality. J Polym Environ. 2024;32:6687–97. 10.1007/s10924-024-03409-z.

[40] Zhang Y, Li R, Chen M, Wang J. Recent advancements in bio-based plasticizers for polylactic acid (PLA): a review. Polym Test. 2024;117:108603. 10.1016/j.polymertesting.2024.108603.

[41] Maghsoud Z, Rafiei M, Famili MHN. Effect of processing method on migration of antioxidant from HDPE packaging into a fatty food simulant in terms of crystallinity. Packag Technol Sci. 2018;31:141–9. 10.1002/pts.2359.

[42] Shi S, Wang M, Wang Z, Qu G, Jiang W, Pan X, et al. Oligomers from the synthetic polymers: another potential iceberg of new pollutants. Environ Health. 2023;1:228–35. 10.1021/envhealth.3c00086. 10.1021/envhealth.3c00086 PMC11504659 39474498

[43] Gorrasi G, Pantani R. Hydrolysis and biodegradation of poly(lactic acid). In: Di Lorenzo ML, Androsch R, editors. Synthesis, Structure and Properties of Poly(lactic acid). Cham: Springer International Publishing; 2017. p. 119–51.

[44] De Luca A, Greco A, Perfetto D, Sepe R. Influence of building position and printing scheme on mechanical properties of fused filament fabrication PLA specimens. Macromol Symp. 2023;411:2300024. 10.1002/masy.202300024.

[45] Kucharczyk P, Pavelková A, Stloukal P, Sedlarík V. Degradation behaviour of PLA-based polyesterurethanes under abiotic and biotic environments. Polym Degrad Stab. 2016;129:222–30. 10.1016/j.polymdegradstab.2016.04.019.

[46] Vikhareva IN, Kruchinina P, Manojlović D. The effect of dicarboxylic acid structure on the plasticizing ability of its ester. Polymers. 2024;16:3372. 10.3390/polym16233372. 39684116 10.3390/polym16233372 PMC11644719

[47] Kang H, Li Y, Gong M, Guo Y, Guo Z, Fang Q, et al. An environmentally sustainable plasticizer toughened polylactide. RSC Adv. 2018;8:11643–51. 10.1039/C7RA13448G. 35542805 10.1039/c7ra13448g PMC9079310

[48] Ludwiczak J, Dmitruk A, Skwarski M, Kaczyński P, Makuła P. UV resistance and biodegradation of PLA-based polymeric blends doped with PBS, PBAT, TPS. Int J Polym Anal Charact. 2023;28:366–82. 10.1080/1023666X.2023.2218696.

[49] Moliner C, Drago E, Lagazzo A, Caputo S, Pettinato M, Finocchio E, et al. Performance of PBS materials with degradable additives for food packaging. J Food Eng. 2026;404:112769. 10.1016/j.jfoodeng.2025.112769.

[50] Capolupo M, Rafiq A, Coralli I, Alessandro T, Valbonesi P, Fabbri D, et al. Bioplastic leachates characterization and impacts on early larval stages and adult mussel cellular, biochemical and physiological responses. Environ Pollut. 2023;319:120951. 10.1016/j.envpol.2022.120951. 36581238 10.1016/j.envpol.2022.120951

[51] Wongphan P, Canellas E, Nerín C, Estremera C, Harnkarnsujarit N, Vera P. Screening and relative quantification of migration from novel thermoplastic starch and PBAT blend packaging. Foods. 2025;14:2171. 10.3390/foods14132171. 40646923 10.3390/foods14132171 PMC12248462

[52] Dimandja J-MD, Archer J, Casanova J, Posenecker E, Stefanuto P-H, Focant J-F, Struk D et al. Development of a standardized protocol for the classification of column sets in comprehensive two-dimensional gas chromatography. LCGC International. 2024;12–20. 10.56530/lcgc.int.kq7288o7

[53] Blomberg J, Schoenmakers PJ, Beens J, Tijssen R. Compehensive two-dimensional gas chromatography (GC×GC) and its applicability to the characterization of complex (petrochemical) mixtures. J High Resolut Chromatogr. 1997;20(10):539–44. 10.1002/jhrc.1240201005.

[54] Mondello L, Cordero C, Janssen HG, Synovec RE, Zoccali M, Tranchida PQ. Comprehensive two-dimensional gas chromatography–mass spectrometry. Nat Rev Methods Primers. 2025;5:7. 10.1038/s43586-024-00379-3.

[55] Labrecque LV, Kumar RA, Dave V, Gross RA, McCarthy SP. Citrate esters as plasticizers for poly(lactic acid). J Appl Polym Sci. 1997;66:1507–13. https://doi.org/10.1002/(SICI)1097-4628(19971121)66:8%3c1507::AID-APP11%3e3.0.CO;2-0.

[56] Guo J, Wu Y, Liu N, Wang L. Effect of acetylated citrate plasticizer on mechanical properties of poly(vinyl chloride). Mater Chem Phys. 2022;287:127068. 10.1016/j.matchemphys.2022.127068.

