# Supplementary material for: Untargeted approaches based on GC-Orbitrap-HRMS and two-dimensional GC–MS for the identification of intentionally and non-intentionally added substances from bio-based food contact materials
Source: Anal Bioanal Chem. 2026 Mar 26;418(10):3099–117. doi: 10.1007/s00216-026-06444-y (PMC13144257; doi:10.1007/s00216-026-06444-y)
Supplement: Supplementary file 1 — Supplementary file1 (DOCX 1.80 MB) [file 216_2026_6444_MOESM1_ESM.docx]

**Untargeted approaches based on GC-Orbitrap-HRMS and two-dimensional GC-MS for the identification of intentionally and non-intentionally added substances from bio-based food contact materials**

Maurizio Piergiovanni ^a*^, Simone Squara ^b^, Marco Fontanarosa ^a^, Cristian Maffezzoni ^a^, Nicolò Riboni ^a^, Antonella Cavazza ^a^, Monica Mattarozzi ^a^, Federica Bianchi ^a^, Michele Suman ^b,c*^, Maria Careri ^a^

^a^ University of Parma, Department of Chemistry, Life Sciences and Environmental Sustainability, Parco Area delle Scienze 17/A, 43124 Parma, Italy

^b^ Barilla G.R. F.lli SpA, Research, Development & Quality, Sensory and Analytical Food

Science, Via Mantova 166, 43100, Parma, Italy

^c^ University of Parma, Department of Food and Drug, Parco Area delle Scienze 27/A, 43124 Parma, Italy

* Corresponding authors: Maurizio Piergiovanni, E-mail address: maurizio.piergiovanni@unipr.it

Michele Suman, E-mail address: michele.suman@unipr.it

Supplementary information

**GC-Orbitrap HRMS data filtering**

Prior to multivariate statistical analysis, features were filtered based on a 5:1 sample/blank ratio and analytical quality (mass accuracy: 5 ppm, peak rating ≥ 6, and RSD% of the peak areas across the QCs after area correction ≤ 20%). The “Peak rating” parameter considers peak shape (Modality, Zig-Zag, Jaggedness, and FWHM to Base), peak area and coefficient of variation. These filters allowed the removal of low-quality peaks, improving the reliability of spectra deconvolution and enabling better annotation. The selected peak rating threshold (≥ 6) in at least 3 runs out of 3 resulted in a good compromise between the number of hits and the analytical quality. In addition, the “Max. Corrected QC Area RSD” filter applied to the QC-corrected area allowed to retain only the features showing stable signal intensity throughout the entire sample set.

**Table S1**. List of analysed bio-based FCM samples and details on the materials and end-use conditions reported on the label.

| Code | Item | Material | End-use indication |
| --- | --- | --- | --- |
| P1 | Cutlery | no-PLA | 70°C, 2h |
| P2 | Cutlery | no-PLA |  |
| P3 | Cutlery | cPLA |  |
| P4 | Cutlery | no-PLA |  |
| P5 | Cutlery | no-PLA |  |
| P6 | Cutlery | no-PLA |  |
| P7 | Cutlery | no-PLA |  |
| P8 | Cutlery | no-PLA |  |
| P9 | Cutlery | cPLA | 70°C, 2h |
| P10 | Cutlery | cPLA |  |
| P11 | Cutlery | cPLA |  |
| P12 | Cutlery | cPLA |  |
| P13 | Cutlery | cPLA |  |
| P14 | Cutlery | cPLA |  |
| PAL1 | Coffee scoop | cPLA |  |
| COP1 | Cup | PLA | 40°C, 2h |
| COP2 | Cup | PLA |  |
| CAN1 | Straw | PLA |  |
| CAN2 | Straw | PLA |  |
| B1 | Glass | PLA |  |
| B2 | Glass | PLA |  |
| B3 | Glass | PLA |  |
| B4 | Glass | PLA |  |
| B5 | Glass | PLA |  |
| B6 | Glass | PLA |  |

**Table S2**. Classification performance of PLS-DA models calculated for both maximum end-use temperature indications and material classification

| Model | Class | Cross - validation | | | External validation | | |
| --- | --- | --- | --- | --- | --- | --- | --- |
|  |  | Sensitivity | Specificity | Precision | Sensitivity | Specificity | Precision |
| Indications of the maximum temperature for end-use | 40°C, 2h | 1.00 | 1.00 | 1.00 | 1.00 | 0.92 | 0.90 |
|  | 70°C, 2h | 1.00 | 1.00 | 1.00 | 0.92 | 1.00 | 1.00 |
| Material resistant to moderate temperature | no-PLA | 1.00 | 0.87 | 0.88 | 0.83 | 0.67 | 0.71 |
|  | cPLA | 0.87 | 1.00 | 1.00 | 0.67 | 0.83 | 0.80 |


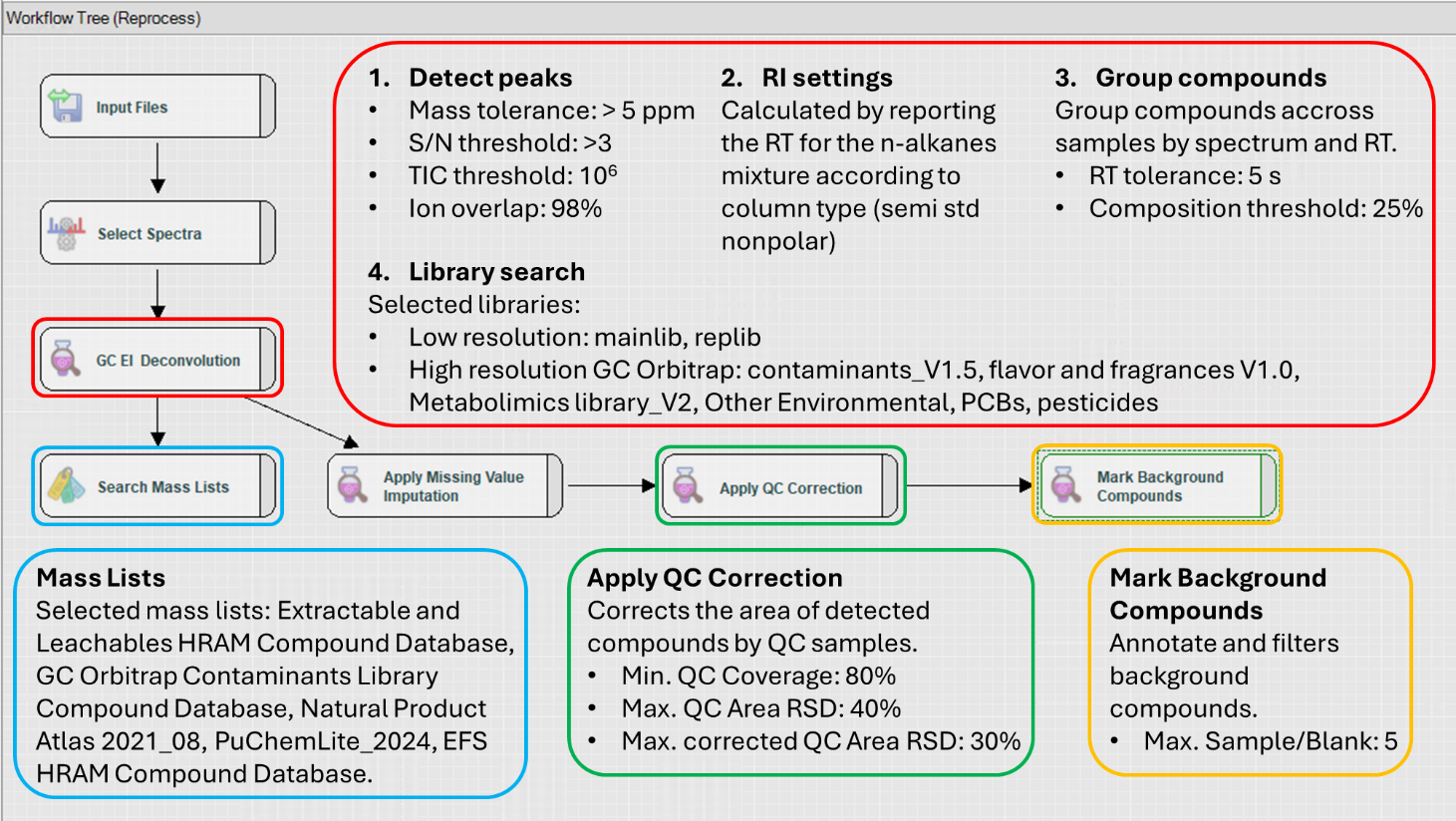


**Fig. S1.** Workflow Tree from the Compound Discoverer 3.0 software displaying GC-Orbitrap HRMS data processing nodes and the associated workflow connections


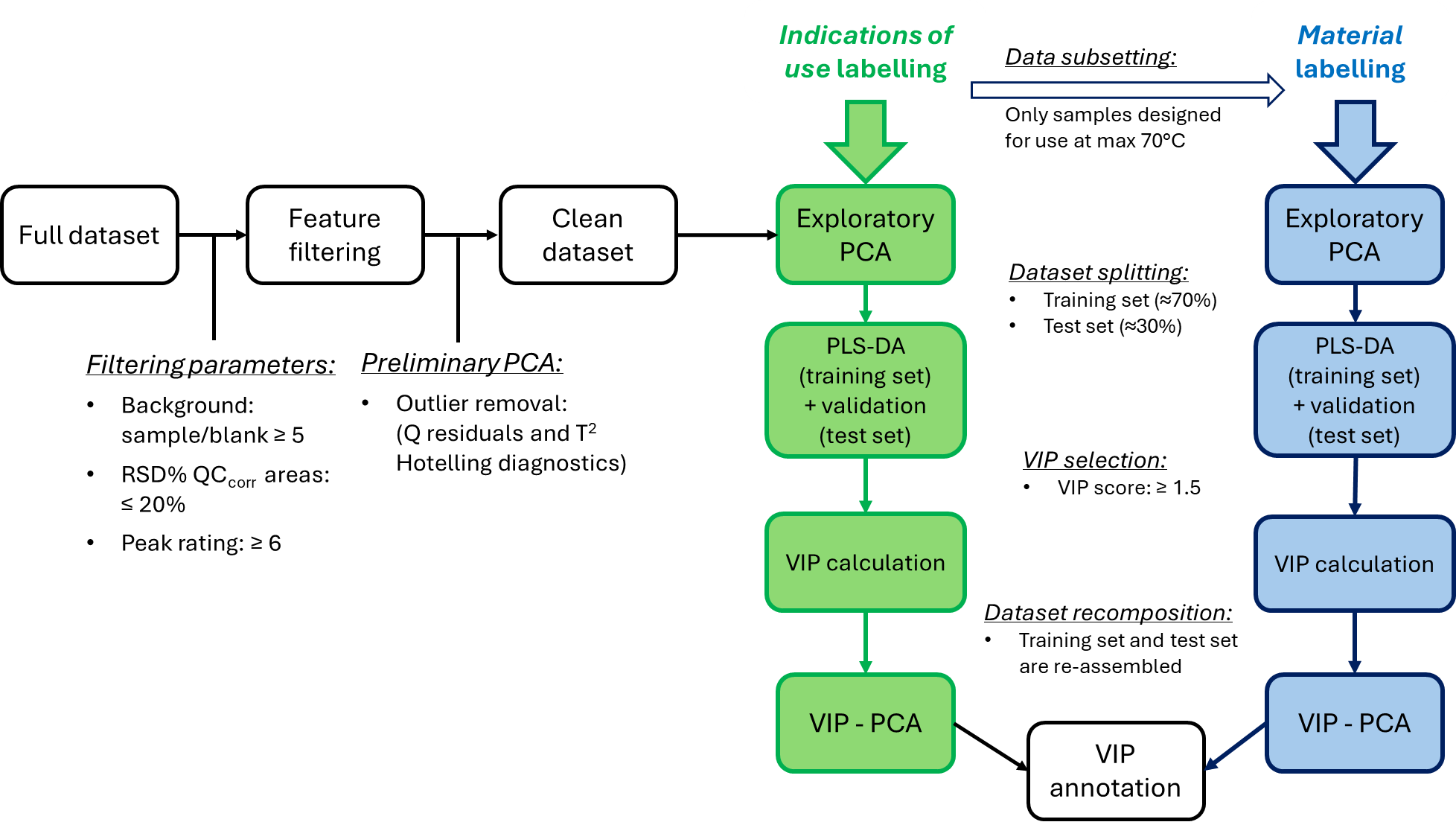


**Fig. S2.** Flowchart of signal processing and extraction of characteristic features for each sample class.


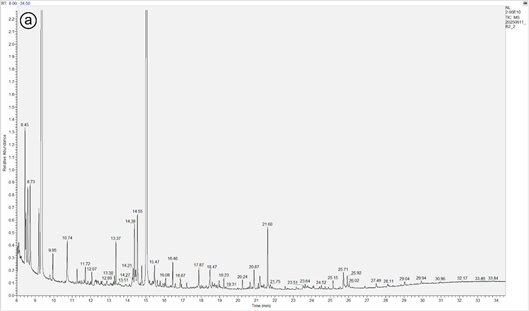

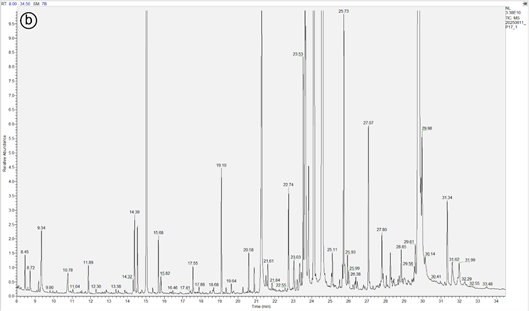

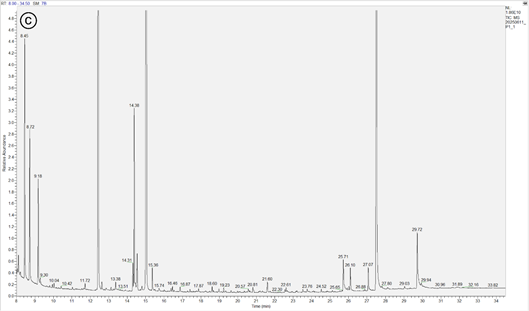


**Fig. S3.** GC-Orbitrap HRMS TIC chromatograms of representative samples of each category of bio-based FCM investigated; (a) B2 (PLA), (b) P14 (cPLA), and (c) P1 (no-PLA).


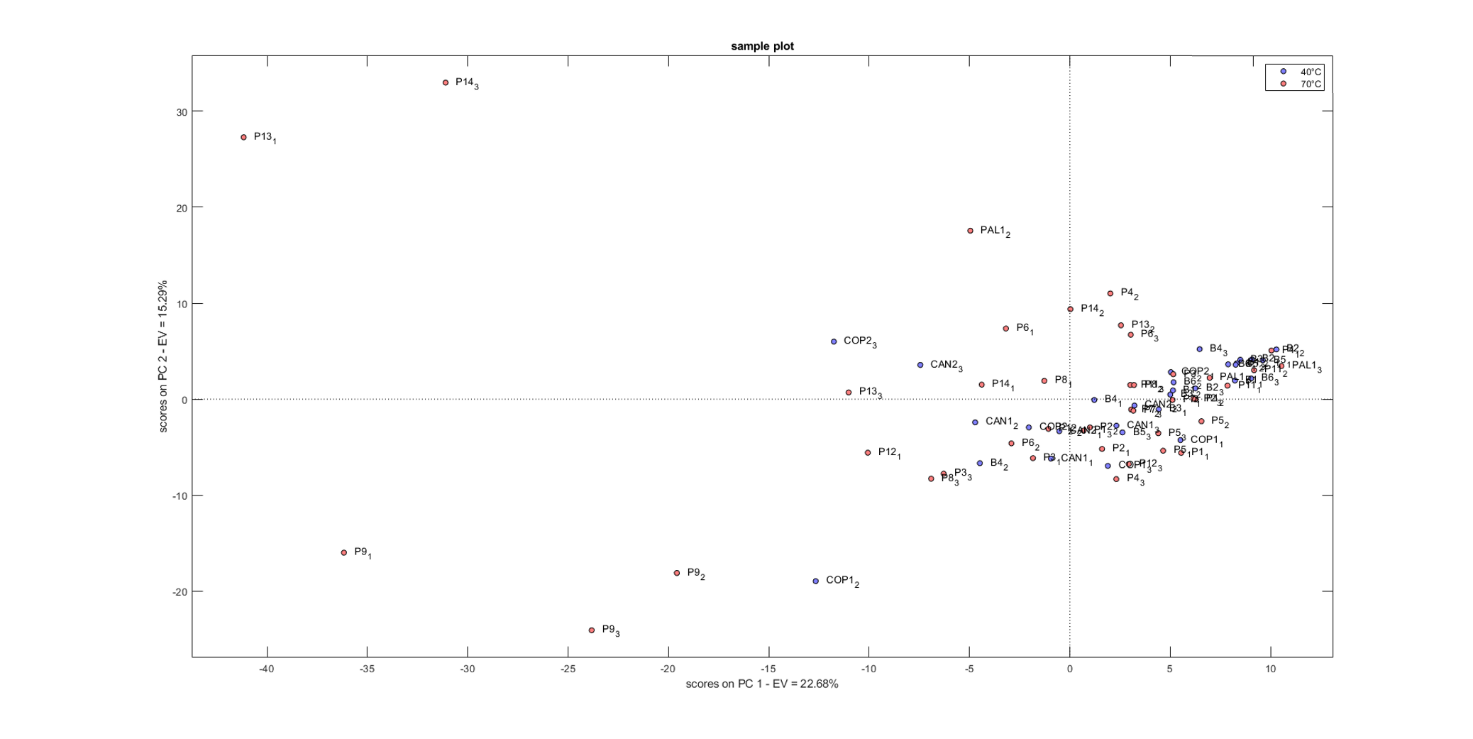


**Fig. S4.** Score plot of the PCA analysis of the clean dataset using all variables. Samples are labelled according to the maximum end-use temperature indications.


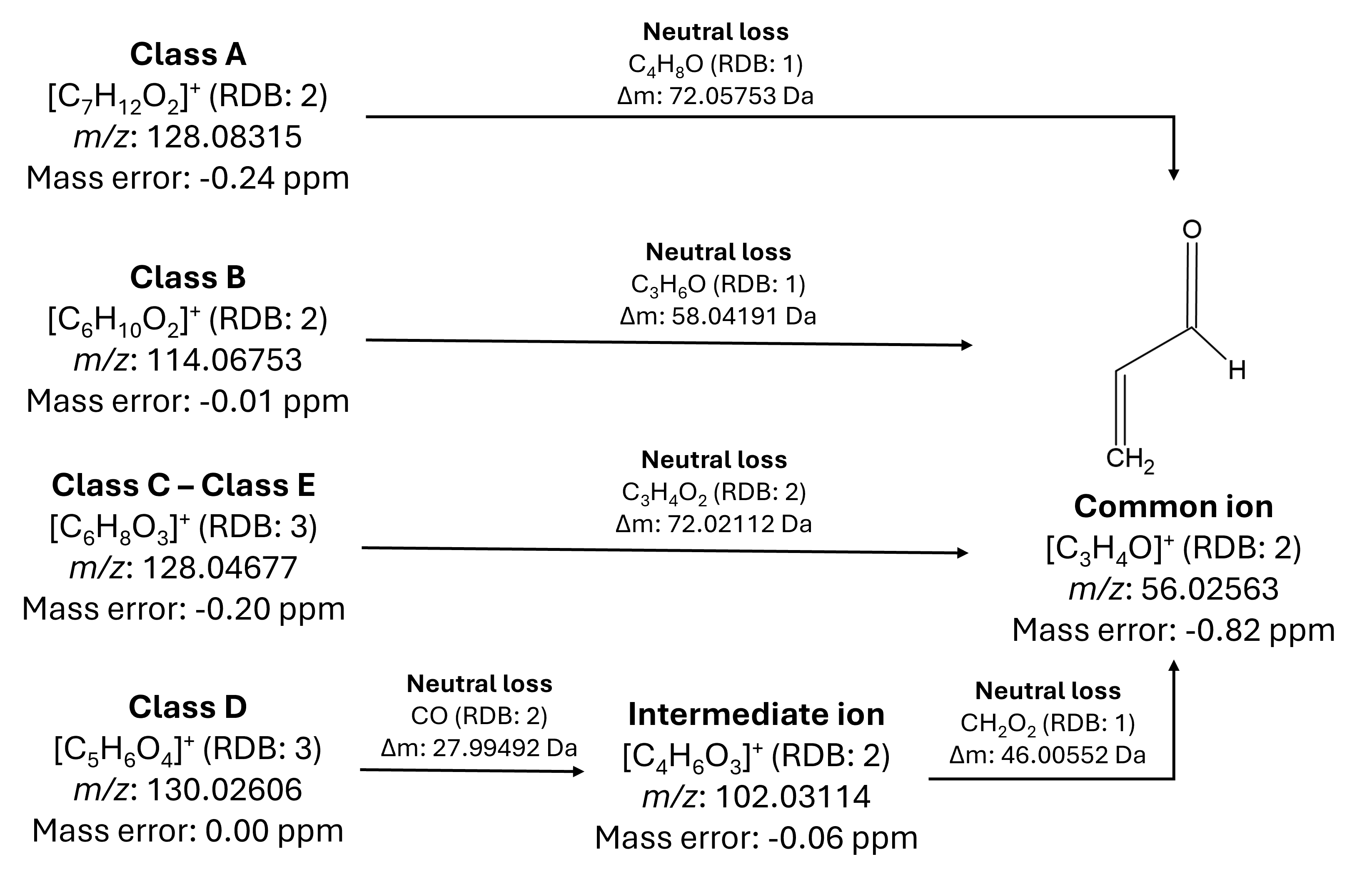
**Fig. S5**. GC-Orbitrap-HRMS EI MS hypothetic rearrangement pattern of Class A, B, C, D, E leading to the formation of the common ion.


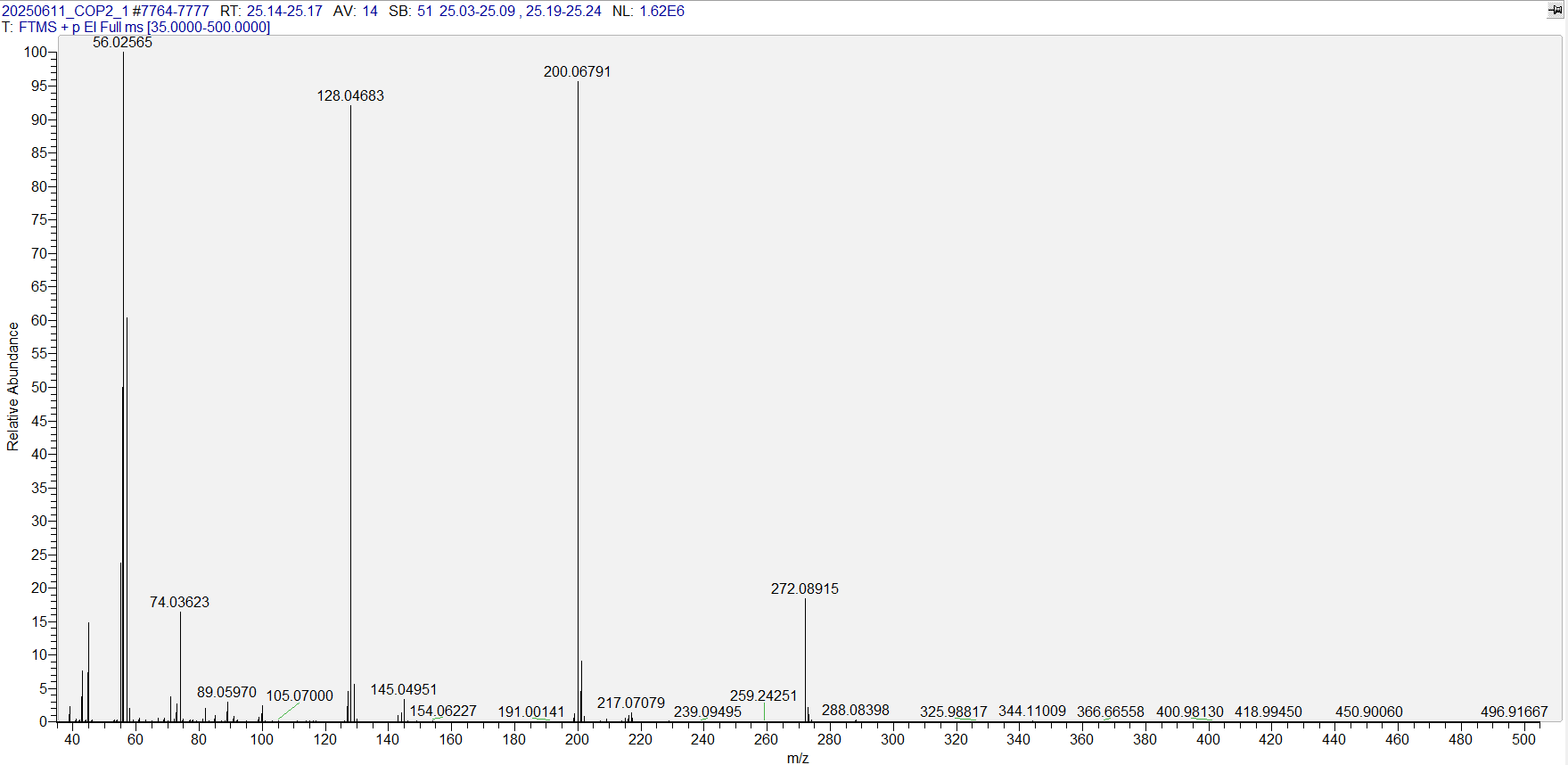


**Fig. S6**. GC-Orbitrap-HRMS EI spectrum of Class C features showing the characteristic *m/z* pattern.

 
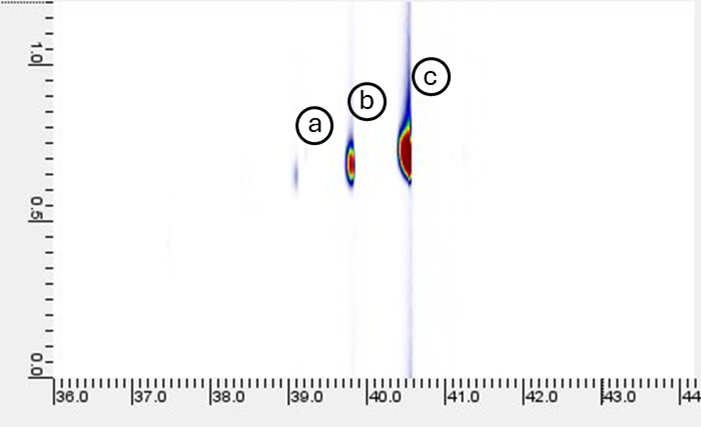


**Fig. S7**. GC×GC-MS extracted ion chromatogram (XIC) of m/z: 259.20 in the cPLA class image showing the butyl-citrate derivatives. Analyte (a) (tR 39.1 min, RI: 2189) was tentatively identified as Butyl Citrate, analyte (b) (tR 39.8 min, RI: 2221) was not identified, analyte (c) (tR 40.5 min, RI: 2269) was tentatively identified as Tributyl Acetylcitrate
